# Monitoring of (Potentially Leukemia-Specific) Immune Cells After AML Relapse Post-Transplantation Across Treatment Groups and Disease Stages

**DOI:** 10.3390/cancers18213421

**Published:** 2026-10-22

**Authors:** Philipp Anand, Julian Stein, Joudi Abdulmajid, Xiaojia Feng, Christian Wichmann, Linping Chen-Wichmann, Christoph Schmid, Helga Maria Schmetzer, Giuliano Filippini Velazquez

**Affiliations:** 1 Dep., Med. III, University Hospital of Munich, 81377 Munich, Germany; 2 Bavarian Cancer Research Center (BZKF), 91054 Erlangen, Germanygiuliano.filippinivelazquez@uk-augsburg.de (G.F.V.); 3 Workgroup: Immunomodulation, Department of Medicine III, University Hospital Munich, Marchioninistraße 15, 81377 Munich, Germany; 4 Department for Transfusion Medicine, Cell Therapeutics and Haemostaseology, University Hospital, Ludwig-Maximilians-Universität München, 81377 Munich, Germany; 5 Department of Haematology and Oncology, University Hospital of Augsburg, 86156 Augsburg, Germany

**Keywords:** AML, leukemia-specific cells, immunomodulation, immune-monitoring, stem cell transplantation, leukemia therapy

## Abstract

**Purpose:** Relapse after allogeneic stem cell transplantation (SCT) in acute myeloid leukemia (AML) remains a major therapeutic challenge. This study longitudinally assessed immune responses in relapsed AML patients post-SCT under different therapeutic regimens, focusing on (potentially) leukemia-specific cytotoxicity and survival outcomes. **Methods:** The composition and profile of peripheral immune cells of 14 AML patients in hematological relapse post-SCT were evaluated by flow cytometry. Patients were stratified by clinical stage and treatment: relapse without therapy (R_w/o_), relapse under/after HMA ± VCLX (R_HMAs/VCLX_), relapse under/after Kit-M treatment (R_Kit-M_), and complete or partial remission after treatment (PR/CR). Frequencies of blasts, dendritic cells, (potentially) leukemia-specific IFNγ-producing or CD107a-degranulating T/NK/CIK cells, and immune checkpoint marker expression (PD-1, CTLA-4, TIM-3, TIGIT, KLRG1) were quantified. Immune profiles were also compared between long-term survivors (‘alive’) and ‘deceased’ patients. **Results:** Increased immune activation, characterized by increased frequencies of (potentially) leukemia-derived dendritic cells, enhanced frequencies of CD107a^+^/IFNγ^+^ effector and memory T/NK cells and reduced frequencies of cells expressing inhibitory checkpoint markers observed in the R_HMAs/VCLX_ group (and even more in a single anecdotal patient, treated with Kit M R_Kit-M_). Patients in remission after treatment (PR/CR) exhibited stabilization of these immune effects, indicating restored immune surveillance. Long-term survivors exhibited higher frequencies of functional CD107a^+^ T and IFNγ^+^ NK/CIK cells, while the ‘deceased’ group displayed signs of increased immune exhaustion and diminished effector responses. **Conclusions:** HMA/VCLX therapy (and Kit-M treatment) induced measurable, potentially) (leukemia-specific) immune responses in relapsed AML patients post-SCT. Elevated functional immune subsets correlated with long-term survival, underscoring their role as potential prognostic biomarkers. Despite a very heterogeneous patient cohort and treatment, our findings support immune monitoring post-SCT to improve risk stratification and highlight the therapeutic potential of immune-modulating treatments in relapsed AML.

## Simple Summary

Relapse after allogeneic stem cell transplantation (SCT) in acute myeloid leukemia (AML) remains a major therapeutic challenge. This study longitudinally assessed immune responses in relapsed AML patients post-SCT under different therapeutic regimens, focusing on (potentially) leukemia-specific cytotoxicity and survival outcomes. Our findings emphasize the critical role of (potentially leukemia-specific) adaptive and innate immune responses in post-SCT survival, serving as valuable prognostic markers to predict patients’ outcomes at relapse after SCT (related or not related to treatment). Furthermore, our study emphasizes the critical role of both adaptive and innate immune responses in post-HSCT outcomes for relapsed AML patients. Advanced flowcytometric techniques could further refine immune monitoring and detection of residual disease, improving prognostic accuracy and treatment decisions. Treatment strategies, particularly those restoring antileukemic immune functions (e.g., by downregulating escape mechanisms or enhancing (e.g., dendritic cell-mediated, DLI-mediated)) have to be developed or optimized and require further refinement for long-term benefits in future trials. Future studies should validate these markers in larger cohorts to refine post-SCT management and explore their therapeutic potential in relapse prevention.

## 1. Introduction

### 1.1. Emerging Treatment Strategies and Post-Transplant Maintenance in Acute Myeloid Leukemia (AML) 

A critical component of successful AML treatment is effective post-transplant maintenance therapy following allogeneic stem cell transplantation (SCT), to prevent relapse and sustain remission by targeting residual leukemic cells that may persist after SCT. Recent advancements in post-transplantation therapies include the use of donor lymphocyte infusions (DLIs) [1] or hypomethylating agents (HMAs), such as azacitidine and decitabine, often in combination with BCL-2 inhibitors like Venetoclax (VCLX), which have shown promise in reducing relapse rates and improving long-term survival [2]. Additionally, the incorporation of immune checkpoint inhibitors, such as PD-1 (Nivolumab) or CTLA-4 inhibitors (Ipilumab), is being explored to enhance immune surveillance and to reduce the risk of relapse [3]. Furthermore, dendritic cell (DC)-based immunotherapies with adoptively transferred manipulated DC or in vivo produced leukemia-derived DC using Kit-M (GM-CSF + PGE1) are gaining attention as a promising approach to stimulate the immune system and to enhance anti-leukemic responses following SCT [4,5]. Some of these novel therapies offer the potential for more effective and less toxic options for maintaining remission after SCT, but further clinical trials are needed to determine their optimal use in AML patients.

### 1.2. Functions of the Immune System

The immune response to AML is orchestrated by both the innate and adaptive immune systems. The innate immune system, acting as the first line of defense, comprises cells such as monocytes, macrophages, dendritic cells (DC: CD80^+^, CD206^+^), natural killer (NK: CD3^−^CD56^+^) cells, cytokine-induced killer (CIK: CD3^+^CD56^+^) cells, and invariant natural killer T (iNKT: 6b11^+^) cells, and their subtypes [6]. Abbreviations for cell subtypes are given in Table 1. DCs, through antigen presentation, bridge the innate and adaptive immune responses [7]. Fast-acting NK cells, along with CIK and iNKT cells, detect and eliminate tumor cells [8,9].

The adaptive immune system involves (potentially leukemia-specific) B and T cells (CD3^+^), with T cells further subdivided into central memory T cells (Tcm/T and cells: CD3^+^CD45RO^+^CD197^+^), effector T cells (Tem/T and cells: CD3^+^CD45RO^+^CD197^−^), ‘antitumor-directed’ Integrinβ7 coexpressing T cells (TIntβ7/T and cells: CD3^+^Intβ7^+^) or γδ T cell receptor T cells (TCRγδ/T and cells: CD3^+^TCRγδ^+^) and regulatory T cells (Treg/T and cells: CD3^+^CD4^+^CD25^++^CD127^(+)^) [20]. Central memory T cells ensure long-term immunity [28], effector T cells actively combat tumor cells, while Treg cells suppress immune responses to prevent autoimmunity, but also inhibit the function of effector T cells and NK cells, to destroy leukemic cells [29,30]. The delicate balance between activation and regulation within the adaptive immune response is crucial, as dysregulation can contribute to immune escape by leukemia cells. Abbreviations for cell subtypes are given in Table 1.

In AML, the immune system’s ability to recognize and eliminate malignant cells is often impaired, which contributes to disease progression and treatment resistance.

### 1.3. Mechanisms of Immune Evasion

AML blasts often exploit immune evasion strategies, dampening the activity of immune cells, which is crucial for their persistence and progression [31]. One prominent strategy involves the upregulation of immune checkpoint markers (ICM) on leukemia and immune cells [32]. These ICM markers, such as PD1, CTLA-4, 2B4, TIGIT, TIM3, and KLRG1, play essential roles in immune modulation by inhibiting the activation and function of effector T cells (TICM/T and cells, e.g., CD3^+^CD279^+^, CD3^+^CD152^+^, CD3^+^CD2B4^+^, CD3^+^TIGIT^+^, CD3^+^TIM3^+^, CD3^+^KLRG1^+^), natural killer cells (NKICM/NK and cells, e.g., CD3^−^CD56^+^CD279^+^, CD3^−^CD56^+^CD152^+^, CD3^−^CD56^+^CD2B4^+^, CD3^−^CD56^+^TIGIT^+^, CD3^−^CD56^+^TIM3^+^, CD3^−^CD56^+^KLRG1^+^) and cytokine-induced killer cells (CIKICM/CIK and cells, e.g., CD3^+^CD56^+^CD279^+^/CD3^+^CD56^+^). PD-1, present on activated T cells, interacts with PD-L1, which is upregulated in response to inflammation and by tumor cells. This interaction inhibits immune activation, leading to T cell exhaustion and impaired antitumor function [33]. CTLA-4, 2B4 and TIGIT are immune inhibitory receptors expressed on T cells and NK cells, thereby allowing leukemia blasts to escape immune surveillance [14,15,23]. Similarly, the expression of TIM3 and KLRG1 on immune cells further contributes to immune suppression by promoting T cell exhaustion and impeding the effective targeting of tumor cells [17,26].

The interplay between these checkpoint markers and regulatory T cells forms a complex immune microenvironment that hinders the effective immune response against leukemia, making immune checkpoint inhibition a promising therapeutic approach to reverse immune suppression and improve outcomes in AML patients.

### 1.4. Immune Profiling as a Prognostic Indicator

Emerging treatment strategies for AML focus on (personalized) immunotherapeutic, hypomethylating, or mutation-based approaches to improve patients’ outcomes. In this context, immune monitoring has become a crucial tool for assessing immune responses and guiding therapies. Qualitative and quantitative evaluation and monitoring of blast and immune cell (sub-)populations could help to evaluate the immune response, detect dysfunction, and to predict relapses or the quality of remissions [34,35].

Leukemia-specific assays allow the analysis and quantification of the (potentially) leukemia-specific/antileukemic immune response, offering insights into the immune system’s ability to target and eliminate leukemic blasts. Especially after stimulation with leukemia-associated antigens (e.g., WT1, PRAME), assays like degranulation (e.g., CD107a) and intracellular cytokine staining (e.g., IFNγ) evaluate (potentially leukemia-specific) immune cell activity. IFN-γ analysis, crucial for macrophage activation and the function of cytotoxic T cells and NK cells, serves as an indicator of (potentially leukemia-specific) immune responsiveness against leukemic cells [21,36]. Therefore, elevated frequencies of IFN-γ-producing immune cells may indicate a more effective immune surveillance mechanism and enhanced tumor control [36].

Similarly, analysis of lysosome-associated membrane glycoproteins (LAMPs) like CD107a detects degranulation, marking the (potentially leukemia-specific) cytotoxic activity of NK cells and cytotoxic T lymphocytes essential for leukemic cell destruction after leukemic antigen stimulation (using WT1 and PRAME [10,37]).

The ICM (CTLA-4, PD-1, TIGIT, and TIM-3) expressing T/NK cells, responsible for immune exhaustion and dysfunction, contribute to immune evasion of leukemic blasts. Monitoring and quantification of these potentially helps to detect relapse or treatment resistance [38,39].

Overall, immune monitoring enhances the understanding of immune dynamics in AML, guiding relapse prediction and optimizing treatment strategies for improved patient outcomes.

### 1.5. Aims

The aims of this study are:

(1) To quantify and characterize (changes in) immune cell populations during different disease phases following SCT in AML patients under varying treatment regimens.
(2) To evaluate (potentially leukemia-specific) immune responses by quantifying IFNγ/CD107a positive immune cell activity.
(3) To correlate frequencies of ICM (e.g., CTLA-4, PD-1, TIGIT, TIM-3) expressing immune cells with disease progression and treatment response in AML patients post-SCT.
(4) To identify potential immune-based prognostic markers by correlating immune profiling data with clinical outcomes, such as relapse risk, sustained remission, and survival.
(5) To generate insights into immune modulation mechanisms that can guide the development of personalized therapeutic strategies and enhance post-SCT monitoring and patient management.

## 2. Material and Methods

### 2.1. Blood Sample Acquisition

Blood samples were obtained with patients’ written informed consent, adhering to the Declaration of Helsinki and approved by the local ethics board (Pettenkoferstraße 8a, 80336 Munich, Ludwig-Maximilians-Universität; ref. no. 33905). The University Hospital Augsburg conducted the blood sampling using 7.5 mL lithium heparin tubes (SARSTEDT AG & Co. KG, Nümbrecht, Germany).

### 2.2. Characteristics of the Patient Cohort

In our longitudinal study, we included a total of 14 AML patients after SCT with a mean age of 59.1 ± 11.8 years, including 9 female and 5 male patients. At the beginning of the study, twelve patients were studied at the relapse stage, one in partial remission, and one in complete remission after treatment of relapse. The observation periods ranged from 10 to 1360 days, with a mean duration of 619 ± 507 days. During the study, seven patients succumbed to the disease. Longitudinal assessments were stratified based on disease status and treatment regimen into the following groups: relapse without therapy (R_w/o_), relapse under/after HMAs/VCLX treatment (R_HMAs/VCLX_), a single relapsed patient under/after Kit-M treatment (R_Kit-M_), partial remission (PR) and complete remission (CR). At relapse, patients showed, on average, 36% blasts in bone marrow (range 0–90%) and 12% in peripheral blood (range 1–75%). During partial remission, blast counts were 4% in bone marrow and 1% in peripheral blood. Overall chimerism at relapse had a mean of 60% (range 6–100%), and T cell chimerism had a mean of 89% (range 46–100%). During partial remission, overall chimerism was 94% and T cell chimerism 88%.

For subsequent survival-based analyses, patients were stratified according to their survival status during follow-up into two outcome groups: long-term surviving (‘alive’: n = 6), comprising patients who were still alive at the last available clinical follow-up, and ‘deceased’ (n = 5), comprising patients who died during the observation period. To obtain a more homogeneous cohort, only patients who were in relapse at the time of the first analysis were included. In addition, patient 1664 was excluded because this patient received Kit-M treatment, which could have introduced a specific treatment-related effect. Thus, 11 patients were included in the survival-based comparison. For these patients, all available longitudinal flow-cytometric measurements obtained during follow-up were assigned to the respective outcome group and analyzed without patient-wise pooling, in order to retain changes occurring during different disease and treatment phases. This grouping was applied in order to explore potential prognostic immune signatures. Further details are provided in Table 2 and Table 3.

### 2.3. Flow Cytometric Analysis for Cell Characterization

The characterization and quantifications of various blast, dendritic cells, monocytes and different immune cell populations, including checkpoint marker (e.g., CD279, CD152, 2B4, TIGIT, KLRG1 and TIM3) expressing subsets was carried out using monoclonal antibodies conjugated with fluorochromes, including FITC (fluorescein isothiocyanate), PE (phycoerythrin), PE/Cy7 (phycoerythrin/cyanine 7), and APC (allophycocyanin) and respective isotype controls and evaluation of data by flow cytometry on a FACSCalibur™ flow cytometer, as shown before [10]. Data analysis was performed with CellQuest software (version 5.1, Becton Dickinson, Heidelberg, Germany). These antibodies were sourced from Beckman Coulter (Krefeld, Germany), Becton Dickinson (Heidelberg, Germany), BioLegend (Amsterdam, Netherlands), Miltenyi Biotec (Bergisch Gladbach, Germany), and Santa Cruz Biotechnology (Heidelberg, Germany). Flow cytometric analyses were performed using a stepwise gating strategy. First, lymphocytes or non-lymphocytes were identified in FSC/SSC plots to allow separate quantification of lymphoid cells versus non-lymphoid cells. Within these populations, defined immune subsets were gated (e.g., CD3^+^ T cells, CD3^−^CD56^+^ NK cells, CD3^+^CD56^+^ CIK cells). In a subsequent step, subgating was applied to further delineate subtypes such as non-naïve T cells and ICM-expressing T cells. Frequencies of defined cell populations were quantified compared to isotype controls and were analyzed relative to the whole gate (e.g., lymphocyte gate) and to a ‘parental population (e.g., IFNγ^+^CD3^+^ in the CD3^+^ cell fraction), since frequencies expressed relative to all cells may be influenced by differences in parent population size. Analyses with less than 30 cells in the respective gates were excluded from further evaluations. Some examples of gated cells are given in Supplementary Figure S8. All analyzed cell subsets are given in Table 1; the panel list is in Supplementary Figure S1.

### 2.4. Detection and Quantification of (Potentially) Leukemia-Specific Cells via Intracellular IFN-γ Staining and Degranulation Assay (DEG)

INZ and DEG assays were conducted to evaluate and quantify the potentially leukemia-specific IFN-γ-producing and CD107a-expressing cells in the peripheral blood (PB) of AML patients during follow-up assessments. Leukemia-associated antigens (LAAs) such as Wilms tumor-1 (WT1) and the preferentially expressed antigen of melanoma (PRAME) were utilized to further stimulate potentially leukemia-specific immune cells. These LAAs can effectively activate targeted immune responses in AML, presenting potential therapeutic strategies for boosting leukemia-specific immunity [40].

Intracellular cytokine staining (INZ) enables the simultaneous detection of immune cell phenotype and intracellular cytokines (e.g., IFNγ) at the single-cell level and therefore allows the identification and quantification of leukemia-specific T (e.g., T_107a_/T and cells: CD107a^+^CD3^+^, T_IFNγ_/T and cells: IFNγ^+^CD3^+^), NK (e.g., NK_107a_/NK and cells: CD107a^+^CD3^−^CD56^+^, NK_IFNγ_/NK and cells: IFNγ^+^CD3^−^CD56^+^), or CIK (e.g., CIK_107a_/CIK and cells: CD107a^+^CD3^+^CD56^+^, CIK_IFNγ_/CIK and cells: IFNγ^+^CD3^+^CD56) cell subsets that mount an anti-leukemic cytokine response (all analyzed (potentially) leukemia-specific subset are given in Table 1). In parallel, the degranulation assay (DEG) measures the cytotoxic potential of immune cells by detecting surface expression of CD107a, a functional marker of lysosomal exocytosis, as shown before [10,21].

### 2.5. Statistical and Graphical Analysis

Flow cytometry data were analyzed using BD CellQuest™ Pro Software (version 5.1, Becton Dickinson, Heidelberg, Germany). Graphical representations of the data were generated utilizing GraphPad Prism 10 software. Results are expressed as the mean ± standard deviation (SD). For statistical evaluation, the nonparametric Kruskal–Wallis test and Mann–Whitney U test were applied, with analyses performed using GraphPad Prism 10 and Microsoft Excel. A *p*-value ≤ 0.005 was deemed “highly significant” (***), a *p*-value ≤ 0.05 was considered “significant” (**), and a *p*-value ≤ 0.1 was regarded as “numerical trend” (*). *p*-values > 0.1 were interpreted as “not significant.” In our exploratory setting, we used the category “numerical trend” (*p*-value between 0.05 and ≤ 0.1) to highlight emerging trends in our small and heterogeneous cohort. We maintained the conventional threshold for statistical significance (*p* ≤ 0.05). The robustness of our statistical findings was confirmed and the potential inflation of type I error due to multiple subgroup comparisons accounted applying additionally Kruskal–Wallis tests with Dunn’s post hoc correction in GraphPad Prism (adjusted *p*-values, *p*_adj). The adjusted *p*-values confirmed the same overall significance pattern as the original results, while non-significant trends remained unchanged. As the correction for multiple testing did not materially affect any of the outcomes, adjusted *p*-values are not shown separately, and the original values are reported throughout for consistency and clarity.

In the follow-up assessments, we stratified results of AML patients into four stage groups based on disease progression and treatment status (Table 2 and Table 3): relapse without therapy (R_w/o_), relapse during treatment with HMAs and/or Venetoclax (R_HMAs/VCLX_), one relapsed patient under Kit-M therapy (R_Kit-M_, Supplementary Table S1), and partial/complete remission (PR/CR). If multiple measurements were taken for a single patient within the same *stage* group under the same *treatment*, the mean values were used for statistical analysis.

## 3. Results

We quantified (potentially leukemia-specific) immune cell dynamics at various stages of the disease and across different treatment regimens by performing statistical evaluations of the frequencies of immune cell populations within four groups (Table 4). Descriptions of immune subsets are given in Table 1. We also aimed to evaluate the predictive relevance of immune cell subsets with respect to patients’ clinical outcomes by comparing immune cell populations of later deceased patients and long-term survivors.

### 3.1. Composition of Blood Cells in AML Patients in Different Stages or Treatment Groups

In the follow-up assessments, we stratified results of AML patients into four stage groups based on disease progression and treatment status (Table 2 and Table 3): relapse without therapy (R_w/o_), relapse during treatment with HMAs and/or Venetoclax (R_HMAs/VCLX_), a single relapsed patient under Kit-M therapy (R_Kit-M_, Supplementary Table S1), and partial/complete remission (PR/CR). If multiple measurements were taken for a single patient within the same stage group under the same treatment, the mean values were used for statistical analysis. Specific criteria for these groups are detailed in Table 4.

#### 3.1.1. Composition of Myeloid Cells (Blast and DC Subtypes)

As anticipated, the analysis of blasts (%Bla/cells) revealed a significant difference (p_Kw_ < 0.0265) between the R_w/o_ (6.8 ± 5.2%, *p* = 0.0336) and R_HMAs/VCLX_ (10.4 ± 6.7%, *p* = 0.0242), compared to the PR/CR (0.8 ± 0.6%) groups (Figure 1a).

In addition to the blast cell analysis, dendritic cells (%DC/cells) and leukemia-derived dendritic cells (%DCleu/cells) were evaluated as part of the myeloid cell profiling. The R_w/o_, R_HMAs/VCLX_ and PR/CR groups exhibited minimal variations in %DC/cells, with an overall mean of 4.6 ± 0.4% across all groups. Likewise, %DCleu/cells (1.4 ± 0.6%) remained relatively consistent across the three groups. In contrast, the only patient treated with Kit-M (R_Kit-M_) displayed substantially elevated levels of both %DC/cells (12.0) and %DCleu/cells (8.1) (Figure 1b,c and Figure S1b,c).

#### 3.1.2. Composition of (Potentially Leukemia-Specific) Adaptive Cells of the Immune System

##### Composition of T Cells

The principal components of the adaptive immune response are T cells and their various subpopulations. In the context of the follow-up analysis of these cell groups, lower frequencies of T cells (%T/cells) were observed in the R_w/o_ (11.6 ± 12.4%) and R_HMAs/VCLX_ (9.1 ± 3.3%) groups relative to the PR/CR groups (13.1 ± 11.3%). A notable exception was observed in the one patient treated with Kit-M (R_Kit-M_), which exhibited a substantial increase in %T/cells (28.3%), compared to the other groups (Figure 2a and Figure S2a).

Additionally, the following T cell subpopulations were analyzed during the course of the study: non-naive (%Tnn/cells), central memory T cells (%Tcm/T), Integrin-β7 (%TIntβ7/cells) T cells and γδ-T cell receptor (%TCRγδ/cells). The analysis of %Tnn/cells (Figure 2b) revealed a comparable distribution across all groups (5.9 ± 1.3%), with the exception of the one patient treated with Kit-M (R_Kit-M_, 18.0%, Supplementary Figure S2b). In contrast, a notable positive trend was observed in the evaluation of %Tcm/T (Figure 2c), with a significant increase from R_w/o_ (4.0 ± 4.5%) to PR/CR (13.1 ± 11.0%) (*p* = 0.0121). The one patient treated with Kit-M (R_Kit-M_) also exhibited a marked increase in %Tcm/T: 18.8%, highlighting a distinct immunological response in this group (Supplementary Figure S2c).

Similar trends were observed for %TIntβ7/cells and %TCRγδ/cells (Figure 2d,e), with elevated values observed in the PR/CR group (TIntβ7: 2.7 ± 1.8%, TCRγδ: 2.1 ± 1.5%), highest values in the one patient treated with Kit-M (TIntβ7: 5.6%, TCRγδ: 10.6%, Supplementary Figure S2d,e) and generally lower values in R_w/o_ (TIntβ7: 1.8 ± 1.8%, TCRγδ: 1.8 ± 1.1%) and R_HMAs/VCLX_ (TIntβ7: 2.1 ± 1.4%, TCRγδ: 1.3 ± 0.9, Figure 2d,e).

##### Composition of Immune Checkpointmarker (ICM) Expressing T Cells

We quantified immune checkpoint marker (ICM) expressing T cells to assess the effects of different therapies and disease progression. The examined ICMS-expressing cells included CTLA-4 (%T152/T), PD-1 (%T279/T), TIM-3 (%TTIM3/T), KLRG1 (%TKLRG1/T), 2B4 (%T2B4/T), and TIGIT (%TTIGIT/T).

%T 152/T and %T 279/T expression (Figure 3a,b) was lowest in the one patient treated with Kit-M (R_Kit-M_) (T152: 15.2%, T279: 26.4%, Supplementary Figure S3a,b), while a largely homogeneous distribution was observed across the other groups (T152: 48.6 ± 3.1%, T279: 42.8 ± 2.6%), with the exception of %T152/T in R_HMAs/VCLX_ (33.9 ± 23.2%). A significant trend (p_KW_ = 0.0173) was observed in the frequencies of %TTIM3/T (Figure 3c), with significantly elevated frequencies across all relapse groups (R_w/o_: 26.8 ± 22.2%, *p* = 0.0099; R_HMAs/VCLX_: 17.1 ± 15.7%, *p* = 0.0302; R_Kit-M_: 38.1%) compared to the PR/CR (7.6 ± 5.4%) group (Figure 3c and Figure S3c). No distinct pattern was evident for %TKLRG1/T (Figure 3d), although frequencies were significantly higher (*p* = 0.0157) in PR/CR (14.8 ± 6.2%) compared to R_HMAs/VCLX_ (7.1 ± 5.3%). In contrast, T2B4/T frequencies (Figure 3e) remained uniformly high across all groups (50.3 ± 9.0%). For frequencies of %TTIGIT/T (Figure 3f), lower frequencies were observed in the relapse groups (mean: 59.5 ± 6.3%) compared to the PR/CR (69.4 ± 24.4%) groups, with a significant difference (*p* = 0.0462) between R_w/o_ and PR/CR. We also analyzed the frequencies of ICM-expressing T cells in all cells (=%TICM/cells: T152/cells, T279/cells, TTIM3/cells, TTIGIT/cells, TKLRG1/cells, and T2B4/cells). No significant differences in ICM-expressing T cells across all subtypes were found in the groups compared.

##### Composition of (Potentially) Leukemia-Specific T Cells

A key aspect of this study was assessing the (potentially) leukemia-specific immune response by quantifying IFNγ- and CD107a-expressing T cells. %T_IFNγ_/cells (Figure 4a) showed slightly elevated frequencies in PR/CR (4.5 ± 3.0%) compared to R_w/o_ (3.3 ± 1.6%), with a marked increase in R_HMAs/VCLX_ (6.4 ± 5.0%). Leukemia-specific subpopulations Tnn and Tcm followed a similar pattern, with minor differences in frequencies of IFNγ (Figure 4b,c). The analysis of leukemia-specific subsets TIntβ7 and TCRγδ revealed the lowest %TIntβ7_IFNγ_/TIntβ7 (Figure 4d) frequencies in R_w/o_ (10.1 ± 4.5%) compared to the other groups (mean: 24.2 ± 3.1%). Degranulating T cells (%T_107a_/cells) exhibited (Figure 4e) higher frequencies in PR/CR (5.9 ± 8.2%), compared to the other groups (R_w/o_: 2.2 ± 2.3%, R_HMAs/VCLX_: 3.8 ± 3.2%). As expected, the one patient treated with Kit-M (R_Kit-M_) deviated from this trend, showing the highest frequencies of degranulating T cells (%T_107a_/cells: 13.8%, Figure S4e). Other degranulating subpopulations (Figure 4f,g and Figure S4f,g) reflected %T_107a_/cells patterns, with slightly increased frequencies in PR/CR (Tnn_107a_: 1.8 ± 2.2%; Tcm_107a_: 0.6 ± 1.0%) and highest frequencies in the only patient treated R_Kit-M_ (Tnn_107a_: 8.6%, Tcm107a: 2.1%). The overall differences in %TIntβ7_107a_/cells (Figure 4h and Figure S4h) frequencies were minimal across all groups, with values ranging from 0.7% to 0.9%, except for the only patient treated with Kit-M (R_Kit-M_), who exhibited the highest frequencies (2.5%). A similar pattern was observed for %TCRγδ_107a_/cells (Figure 4i and Figure S4i), where frequencies remained relatively low in most groups (mean: 0.5 ± 0.1%) but peaked under Kit-M therapy (3.4%).

#### 3.1.3. Composition of (Potentially Leukemia-Specific) Innate Cells of the Immune System

We examined NK cells, CIK cells, and iNKT cells, including their subpopulations. Overall, frequencies of %CIK/cells and %NK/cells varied only slightly between groups (Figure 5a,b and Figure S5a,b). The highest %CIK/cell frequencies were detected in the PR/CR group (0.8 ± 1.0%), while the remaining groups showed comparable levels (0.5 ± 0.0%). Similar trends were observed for %NK/cells, displaying largely consistent values across all relapse groups (3.4 ± 0.5%) and showing the highest frequencies in PR/CR (4.4 ± 3.3%). In contrast, iNKT/cells showed greater variability (Figure 5c and Figure S5c), with the lowest frequencies in R_w/o_ (1.3 ± 0.8%) and the highest in the one patient treated with Kit-M (R_Kit-M_, 7.2%).

##### Composition of Immune Checkpoint Marker (ICM) Expressing Cells of the Innate Immune System

We also analyzed ICM-expressing NK and CIK cells to identify potential immune evasion mechanisms or derive insights into their expression patterns. Frequencies of %CIK279/CIK (Figure 6a and Figure S6a) displayed a significant difference between the groups (p_KW_=0.0447), with an increasing trend from R_w/o_ (55.9 ± 20.7%) to PR/CR (78.1 ± 9.5%) and a significant difference between the two groups (*p* = 0.0125). In contrast, frequencies of %NK279/NK (Figure 6b and Figure S6b) exhibited a similar pattern to %T279/T, with relatively consistent frequencies across all groups (mean: 17.9 ± 1.3%), except for a notable decrease observed in the one patient treated with Kit-M (11.0%). %NKTIM3/NK frequencies (Figure 6c and Figure S6c) peaked in R_Kit-M_ (58.6%) and R_HMAs/VCLX_ (50.8 ± 27.1%), while lower frequencies were observed in PR/CR and, unexpectedly, R_w/o_. A decreasing trend was noted for %NKKLRG1/NK (Figure 6d), with the highest levels in R_w/o_ (9.1 ± 8.7%) followed by a decline in the one patient treated with Kit-M (R_Kit-M_, 1.5%) and a decrease (numerical trend: *p* = 0.0580) in PR/CR (3.4 ± 3.9) compared to R_w/o_. %NK2B4/NK (Figure 6e and Figure S6e) remained uniformly high across all groups (69.9 ± 9.5%). %NKTIGIT/NK (Figure 6f and Figure S6f) showed lower frequencies in R_HMAs/VCLX_ (24.9 ± 19.9%), while the remaining groups exhibited consistently elevated frequencies (50.6 ± 4.5%). We also analyzed the frequencies of ICM-expressing NK and CIK cells in all cells (=%NKICM/cells and %CIKICM/cells: NK279/cells, NKTIM3/cells, NKTIGIT/cells, NKKLRG1/cells, NK2B4/cells, and CIK279/cells). No significant differences in ICM-expressing innate immune cells across all subtypes were found in the groups compared.

##### Composition of Potentially Leukemia-Specific Cells of Innate Immune System

To assess the potentially leukemia-specific immune response of the innate immune system, we analyzed IFNγ- and CD107a-positive cells. Both %CIK_IFNγ_/CIK (p_KW_=n.s) and %NK_IFNγ_/NK (p_KW_=0.0207) showed (Figure 7a,b) increased frequencies in the one patient under Kit-M treatment (R_Kit-M_ and in PR/CR. Degranulating %CIK_107a_/cells (Figure 7c and Figure S7c) remained low across all groups, with slight increases in the one patient under Kit-M treatment (R_Kit-M_) and PR/CR. %NK_107a_/cells (Figure 7d and Figure S7d) demonstrated the lowest frequencies in the R_w/o_ therapy group (0.8 ± 0.8%), with a significant rise in the one patient under Kit-M treatment (4.7%), while other groups stayed between 1.5% and 1.9%. A similar trend was seen in %iNKT_107a_/cells (Figure 7e and Figure S7e), with the lowest frequencies in R_w/o_ therapy (0.5 ± 0.7%), and an increase in the one patient under Kit-M treatment (R_Kit-M_, 1.1%) and PR/CR (0.9 ± 1.0%) groups, though overall differences were minimal.

### 3.2. Composition of Blood Cells in Relation to Survival Time: A Comparison Between Long-Term Surviving and Deceased Patients

We classified the patients into two groups based on their survival status during the follow-up period: long-term surviving (‘alive’: n = 6), defined as all patients still alive at the time of the last available clinical follow-up and ‘deceased’ (n = 5), defined as patients who had died during the observation period and compared both groups (‘alive’ vs. ‘deceased’). Only patients in the relapse stage at the time of the 1st analysis were included to ensure a homogeneous cohort. The mean observation period was 619 ± 507 days (range 10–1360 days). Within this timeframe, patients of the ‘alive’ group showed a mean survival of 980 ± 350 days, whereas patients of the ‘deceased’ group had a mean survival of 169 ± 140 days. To avoid bias introduced by differing therapeutic effects, patient 1664, treated with a Kit-M therapy, was excluded from the analysis. All individual measurements from the included patients were considered, without pooling data per patient, to provide insights into the course of the disease, thereby enabling the identification of potential predictive markers or cell frequencies for disease progression. All individual measurements from the included patients were considered, without pooling data per patient, to provide insights into the course of the disease, thereby enabling the identification of potential predictive markers or cell frequencies for disease progression. We deliberately retained the longitudinal measurements because averaging all measurements per patient would have combined samples obtained during biologically distinct disease and treatment phases and could therefore have obscured relevant changes associated with relapse, treatment, or remission. Due to the longer follow-up of the ‘alive’ group, these patients contributed more samples obtained during treatment response or remission, whereas samples from the ‘deceased’ group were predominantly obtained during active relapse. Therefore, this longitudinal comparison should be regarded as exploratory and may reflect differences in disease status, treatment effects, and immune reconstitution in addition to a potential association with survival. We provide, in addition, some patients’ characteristics in the groups compared. The ‘*alive*’ group comprised three female and three male patients with a mean age of 63.2 ± 8.8 years, whereas the ‘*deceased*’ group comprised four female and one male patient with a mean age of 55.6 ± 10.3 years. Mean overall/T cell chimerism at first analysis was 46.0 ± 40.8%/85.2 ± 20.8% in the ‘*alive*’ group and 71.8 ± 20.9%/92.6 ± 7.6% in the ‘*deceased*’ group. Mean peripheral blood blast counts were 4.8 ± 3.2% and 22.6 ± 29.5%, respectively. The mean total number of SCTs was comparable between both groups (‘alive’: 1.8 ± 0.8; ‘deceased’: 1.6 ± 0.5). These differences should be considered when interpreting the immune profiles of both outcome groups.

#### 3.2.1. Comparison of Myeloid Cells (Blast and DC Subtypes)

The analysis of the myeloid cell lineages revealed a notable, yet statistically insignificant (*p* = 0.1093), difference in %Bla/cells (Figure 8) between the ‘deceased’ group (10.1 ± 3.8) and the ‘alive’ group (5.9 ± 6.8). No relevant differences were observed between the groups for both %DC/cells and %DCleu/cells.

#### 3.2.2. Comparison of (Potentially Leukemia-Specific) Adaptive Cells of the Immune System

##### Comparison of (Immune Checkpoint Expressing) T Cells

Comparison of the different T cell populations consistently revealed elevated values within the ‘alive’ group. Notably, significantly higher frequencies of %T/cells (Figure 9a) were seen in the ‘alive’ group (18.6 ± 18.3%) compared to the ‘deceased’ group (5.7 ± 4.2%) (*p* = 0.0138), with moreover potentially significant differences were observed for %TIntβ7/cells (*p* = 0.0544) and %TCRγδ/cells (*p* = 0.0697) in the ‘alive’ vs. ‘deceased’ (Figure 9a–e), and non-significant differences, though with higher values in the ‘alive’ group for %Tnn/cells and %Tcm/cells (‘deceased’—Tnn: 3.1 ± 3.6%, Tcm: 3.2 ± 3.8%; ‘alive’—Tnn: 6.7 ± 8.2%, Tcm: 10.1 ± 14.7%).

In contrast, frequencies of ICM-expressing T cells revealed highly significant differences (*p* = 0.0018) only for %T2B4/T (Figure 9g), with remarkably elevated frequencies of ICM-expressing T cells in the ‘alive’ group (59.4 ± 26.9%) compared to the ‘deceased’ group (28.6 ± 19.8%). %T279/T (Figure 9f) exhibited an opposite trend, albeit with a very minor difference. %T152/T, %TTIM3/T, %TKLRG1/T, and %TTIGIT/T also demonstrated only minimal differences between the groups.

Comparison of the different T cell populations consistently revealed elevated values within the ‘alive’ group. Notably, significantly higher frequencies of %T/cells (Figure 9a) were seen in the ‘alive’ group (18.6 ± 18.3%) compared to the ‘deceased’ group (5.7 ± 4.2%) (*p* = 0.0138). A similar non-significant trend was seen for %Tnn/cells (Figure 5a,b) and %Tcm/cells (Figure 9c), with higher values in the ‘alive’ group (Tnn: 6.7 ± 8.2%, Tcm: 10.1 ± 14.7%) than in the ‘deceased’ group (Tnn: 3.1 ± 3.6%, Tcm: 3.2 ± 3.8%). For %TIntβ7/cells (Figure 9d) and %TCRγδ/cells (Figure 9e), potentially significant differences were observed (%TIntβ7/cells: *p* = 0.0544 and %TCRγδ: *p* = 0.0697), again with elevated values in the ‘alive’ group.

Analysis of inhibitory checkpoint marker (ICM) expression revealed only subtle group-dependent differences for most ICMs. %T279/T (Figure 9f) showed a minor opposite trend between groups, whereas %T2B4/T (Figure 9g) exhibited a highly significant difference (*p* = 0.0018) between the ‘alive’ group (59.4 ± 26.9%) and the ‘deceased’ group (28.6 ± 19.8%). Other ICMs, including %T152/T, %TTIM3/T, %TKLRG1/T, and %TTIGIT/T demonstrated only marginal variation between the groups.

##### Comparison of Potentially Leukemia-Specific T Cells

In the next step, we compared differences in potentially leukemia-specific T cells. Frequencies of %T_IFNγ_/cells (Figure 10a) were higher in the ‘alive’ (5.3 ± 4.2%) vs. the ‘deceased’ (3.3 ± 2.4%) group, while no relevant differences were found in %Tnn_IFNγ_/Tnn, %Tcm_IFNγ_/Tcm, and %TIntβ7_IFNγ_/TIntβ7). CD107a-positive T cells showed significantly higher frequencies of %T_107a_/cells (Figure 10b) in the ‘alive’ vs. the ‘deceased’ group (‘alive’: 9.7 ± 16.0%; ‘deceased’: 3.0 ± 3.7%; *p* = 0.0516). Similarly, frequencies of %Tnn_107a_/cells, %Tcm_107a_/cells, and %TIntβ7_107a_/cells (Figure 10c–e) also showed elevated values in the ‘alive’ cohort, but without reaching statistical significance. By contrast, frequencies of %TCRγδ_107a_/cells (Figure 10f) demonstrated a significant difference with higher values in the ‘alive’ cohort (‘alive’: 0.9 ± 1.4%; ‘deceased’: 0.3 ± 0.3%; *p* = 0.0466).

#### 3.2.3. Comparison of (Potentially Leukemia-Specific) Innate Cells of the Immune System

The second key component of the immune defense is the innate immune system. For %CIK/cells (Figure 11a), a clearly significant difference was observed (*p* = 0.0237), with elevated values in the ‘alive’ group (1.1 ± 1.9%) compared to the ‘deceased’ group (0.2 ± 0.1%). Frequencies of %NK/cells (Figure 11b) revealed (non-significantly) increased values in the ‘alive’ vs. the ‘deceased’ group, but the difference was comparatively smaller and not significant (‘alive’: 3.5 ± 4.3%; ‘deceased’: 3.1 ± 2.5%). %iNKT/cells showed no relevant differences between the groups compared.

The comparison of ICM-expressing CIKs and NK cells differed from that on T cells. Non-significantly lower frequencies of %NK279/NK were seen in the ‘alive’ vs. the ‘deceased’ group (‘alive’: 10.4 ± 12.3%; ‘deceased’: 20.4 ± 22.3%;) and %NKKLRG1/NK (‘alive’: 4.9 ± 6.6%; ‘deceased’: 10.9 ± 11.6%) (Figure 11c,d), while %NK2B4/NK (Figure 11e) showed no significant differences.

Finally, we compared frequencies of potentially leukemia-specific CIK and NK cells (Figure 11f–h). A significant difference with higher values in the ‘alive’ group was observed for both %CIK_IFNγ_/CIK (*p* = 0.0186) and %NK_IFNγ_/NK (*p* = 0.0110) (‘alive’—CIK: 49.7 ± 24.4%, NK: 38.9 ± 22.1%; ‘deceased’—CIK: 23.7 ± 20.0%, NK: 14.0 ± 6.5%). Analysis of CD107a-positive cells revealed relevant differences only for %CIK_107a_/cells (‘alive’: 0.6 ± 1.4%; ‘deceased’: 0.1 ± 0.1%), while %NK_107a_/cells and %iNKT_107a_/cells showed similar values for both groups.

### 3.3. Potentially Leukemia-Specific T Cell Dynamics in the Course of Disease and Treatment in Individual Patients

The longitudinal monitoring of CD107a-expressing T cells in three AML patients of the ‘alive’ group revealed dynamic changes in both %T_107a/_T and %Tnn_107a_/T during the course of the disease.

In patient 1650 (Figure 12a), frequencies of %T107a/T increased from 12.3% at day 0 to 28.9% at day 179 following treatment with hypomethylating agents and Venetoclax during relapse (R_HMAs/VCLX_). Following the second SCT and entry into a second complete remission (CR2), %T_107a_/T transiently rose to 43.0%, peaking further at 71.5% by day 533 after donor lymphocyte infusion (DLI). A marked decline was subsequently observed toward the end of the follow-up period. Frequencies of %Tnn_107a_/T showed a parallel trend, with a slight initial increase to 15.9% during R_HMAs/VCLX_ and a strong rise after the second SCT and donor lymphocyte infusion (DLI).

In patient 1654 (Figure 12b), an initial decline of frequencies of %T_107a_/T was observed during R_HMAs/VCLX_ followed by a steadily increase during the third complete remission (CR3), before strongly declining at the end of our follow-up analysis. Frequencies of %Tnn_107a_/T followed a similar trend, with an initial decrease from 49.7% to 9.4% during R_HMAs/VCLX_, followed by minimal changes during early CR3. In the later follow-up period, a notable expansion was evident.

Patient 1663 (Figure 12c) displayed comparable changes in frequencies of %T_107a/_T and %Tnn_107a_/T, characterized by sustained elevations during the fourth complete remission (CR4) following a third SCT. These increases remained stable over time, consistent with long-term expansion of CD107a-positive T cells in the context of post-transplant immune reconstitution.

## 4. Discussion

### 4.1. Prognosis of and Therapeutic Options for Relapsed AML Patients Post-Hematopoietic Stem Cell Transplantation (HSCT)

Hematopoietic stem cell transplantation (HSCT) remains the most effective curative option for AML [41,42], yet relapse occurs in up to 40% of patients and continues to drive poor prognosis [43,44]. This highlights the urgent need for improved monitoring strategies and novel therapeutic approaches.

Among relapse-directed therapies, hypomethylating agents (with or without Venetoclax) [45], have gained particular relevance, not only because of their clinical efficacy but also due to their immunomodulatory effects on tumor suppressor genes [46,47].

Donor lymphocyte infusion (DLI) represents another promising approach to enhance graft-versus-leukemia (GvL) effects [48,49], although its application is limited by the high risk of graft-versus-host disease (GvHD) [50,51].

In parallel, immune checkpoint therapies are emerging as crucial tools to address immune escape mechanisms in AML [52,53,54]. While dendritic cell–based immunotherapies offer an innovative, low-toxicity treatment modality, with potential for repeated, cost-effective administrations [4,5,22]. Although these approaches show promising activity, their long-term survival benefits remain limited, and a second HSCT—despite being potentially curative in selected cases—continues to be associated with substantial risks [55].

### 4.2. Rationale Behind the Grouping of Study Cohorts (R_w/o_, R_Kit-M_, R_HMAs/VCLX_, PR/CR) Prognostic Relevance and Limitations

To analyze immune responses and disease progression in relapsed AML patients post-SCT, patient samples were categorized based on disease stages and treatments. This stratification aims to detect potential differences in immune reconstitution and dynamics influenced by treatment. Relapsed patients were grouped by therapy: patients with no treatment (R_w/o_), treated with hypomethylating agents with/without Venetoclax (R_HMAs/VCLX_), or (one patient) treated with immune modulatory therapy Kit-M (R_Kit-M_), reflecting distinct impacts of the therapy on leukemic burden and immune response. Patients with PR and CR after SCT relapse were combined in group PR/CR due to their low tumor load and immunological similarities. Within PR/CR, different treatments were analyzed collectively to ensure comparability while maintaining statistical power. Further subdivision would have resulted in excessively small groups, limiting meaningful analysis. This approach enables the best possible evaluation of immune signatures and treatment effects on post-SCT outcomes in our relapsed AML cohorts. The retrospective nature of our analysis, which comprises 14 patients treated under heterogeneous and non-standardized conditions, inherently limits statistical robustness. Nevertheless, the data obtained provide indications of prognostic relevance.

Distinct immune cell subsets appeared to align with particular disease stages and survival and reflected differences in therapeutic modes of action, suggesting potential prognostic utility. Validation of these preliminary observations will require larger prospective studies, ideally randomized clinical trials with standardized treatment regimens and harmonized time points of immune monitoring. Of note, in one patient receiving the novel immunomodulatory compound Kit-M, we detected leukemia-specific activation of effector and memory immune cells [4,5]. Such responses may contribute to clearance of residual blasts and support disease stabilization or maintenance of remission.

Another consideration is that donor- and recipient-derived immune cells were not and could not be systematically distinguished during longitudinal analyses. Given the high overall and T cell chimerism at first analysis after relapse, the majority of the immunological alterations measured are likely to reflect donor-derived immune responses. Still, potential contributions from mixed chimerism may have influenced immune cell variability, which should be more precisely characterized in future studies.

### 4.3. ICM-Expressing Immune Cells in AML: Mechanisms of Immuno-Escape, Relapse, and Therapeutic Implications

#### 4.3.1. Role of Myeloid Cells to Overcome Immuno-Escape

A key aspect of the immune response is the presence of dendritic cells (DCs), which are critical antigen-presenting cells that initiate (antigen-specific) immune responses against malignant cells [56,57]. Leukemia-derived dendritic cells (DCleu) present leukemia-specific antigens, thereby enhancing the immune system’s ability to mount a targeted response against leukemia cells [4,58]. Our study demonstrated that Kit-M (GM-CSF + PGE1) effectively stimulated the generation of (leukemia-specific) dendritic cells in the one anecdotal patient treated in vivo (Figure 1b,c). Additionally, in R_HMAs/VCLX,_ induction of a mild stimulatory effect on the provision of DCleu was also observed (Figure 1c). The low values observed in the PR/CR group may be attributed to the limited sample size. Our results suggest that Kit-M, along with HMAs/VCLX, may contribute to the long-term stabilization of remission through their costimulating or immune-modulating effects, although results should be interpreted cautiously [4,59]. Frequencies of blasts (Figure 1a) were higher in R_w/o_ compared to R_HMAs/VCLX_ and in the one patient treated with Kit M (R_Kit-M_), due to the timing of measurements, as the R_w/o_ group was assessed after relapse detection. This temporal difference limits direct conclusions regarding the efficacy of therapeutic regimens based on blast levels. The relatively high blast frequencies observed in the patient treated with Kit-M (R_Kit-M_, 13.5%) group are likely due to the inclusion of this only single patient, who presented with refractory AML and advanced disease progression.

#### 4.3.2. Role of Cells of the Adaptive Immune System to Overcome Immuno-Escape 

Kit-M treatment of the single relapsed patient also led to elevated (potentially leukemia-specific/potentially antileukemic T cell populations (Tnn, Tcm, TCRγδ, TIntβ7; Figure 2a–e and Figure S2a–e), enhancing antileukemic responses (as indicated by increased CD107a-positive cells (Figure 4d–f and Figure S4d–f) and the provision of immunological memory, particularly through (leukemia-specific) Tcm expansion, as shown before in ex vivo settings [10,60]. This may suggest that Kit-M induces a long-term immunological effect, while also boosting anti-tumor subpopulations like TCRγδ and TIntβ7 positive cells (Supplementary Figures S2d,e and S4h,i), which are critical for tumor surveillance and blast lysis [27,61]. During remission (PR/CR), elevated T cell populations (T, Tcm, TIntβ7) reflect a sustained immune response, actively targeting residual leukemia and establishing an immunological memory (Figure 2a,c,d and Figure 4d,g,h). In contrast, R_w/o_ and R_HMAs/VCLX_ show diminished T cell activation, likely due to immune evasion mechanisms, contributing to disease progression [32,62]. The upregulation of these subsets in R_HMAs/VCLX_ compared to R_w/o_ suggests that these agents could stimulate T cell activation and cytolytic activity [47,63], further supported by the addition of VCLX to enhance T cell cytotoxicity [64]. While Kit-M has previously shown immune-modulatory activity in independent case reports [4,5], the effects observed under HMAs/VCLX in our cohort were less pronounced and might in part also reflect reconstitution of donor hematopoiesis and graft-versus-leukemia activity rather than direct induction of immune responses by chemotherapy.

A central mechanism of immune evasion in AML involves modulation of immune checkpoint molecules (ICMs). In our cohort, %T279/T and %T152/T were reduced in the patient treated with Kit-M (R_Kit-M_) and, to a lesser extent, in R_HMAs/VCLX_, while %TKLRG1/T showed a similar pattern. As these markers are associated with T cell exhaustion [65,66]; ref. [17], their downregulation may reflect improved T cell fitness under these treatments. Frequencies of TIM-3^+^ T cells were lower in PR/CR compared to relapse groups, consistent with its reported role in suppressing T cell activity [26]. In contrast, 2B4^+^ and TIGIT^+^ T cells did not follow a uniform pattern associated with immune exhaustion, suggesting a context-dependent role of these markers. Given their dual stimulatory and inhibitory functions [23,25,67], their exact contribution to T-cell regulation and leukemic immune evasion in this setting remains uncertain. Overall, our data suggest that HMAs (+/− VCLX) and probably also Kit-M [4,5] may influence ICM expression and thereby modulate T-(immune)-cell responses [65], though this requires confirmation in larger studies.

#### 4.3.3. Role of Cells of the Innate Immune System to Overcome Immuno-Escape 

The innate immune system is crucial for early detection and response to malignancies, including acute myeloid leukemia (AML). Key components such as NK cells, CIK cells, and iNKT cells are essential for immune surveillance and tumor elimination [68,69,70]. Our analysis following relapse treatment after stem cell transplantation (SCT) revealed distinct patterns of immune cell distribution, including ICM-expressing cells, indicating complex immune evasion and modulation by therapies.

Frequencies of CIK/cells and NK/cells exhibited minimal variations between the groups (Figure 5a,b), with highest frequencies in PR/CR, suggesting a link between immune cell frequencies and remission status. Frequencies of iNKT/cells displayed greater variability in treatment groups (Figure 5c and Figure S5c), with increases in the patient treated with Kit-M (R_Kit-M_) and R_HMAs/VCLX_, possibly indicating a therapeutic impact of HMAs and Kit-M on the provision of iNKT immune components [18]. The lowest frequencies were observed in the R_w/o_ group, which aligns with previous studies highlighting the critical role of iNKT cells in anti-tumor immunity [68]. Potentially leukemia-specific responses (frequencies of IFNγ-producing cells), including frequencies of %NK_IFNγ_/NK and %CIK_IFNγ_/CIK (Figure 7a,b and Figure S7a,b), showed increased frequencies in the Kit-M treated patient, as well as in CR/PR, which could point to an induced potentially antileukemic response in these groups [71]. A similar trend was observed in frequencies of potentially leukemia-specific (degranulating, CD107a^+^) CIK cells (Figure 7c), reflecting enhanced degranulation and cytotoxic potential in remission. Frequencies of NK_107a_/cells (Figure 7d) were lowest in R_w/o_, increased in R_HMAs/VCLX_ and PR/CR, and peaked in the patient treated with R_Kit-M_, suggesting improved induced NK cytotoxicity in these patients, which is crucial for AML blast elimination and supports the importance of NK cells in AML immunity [11,72]. Frequencies of iNKT_107a_/cells were low in all treatment groups, but highest in the single patient treated with Kit-M (R_Kit-M_) and the PR/CR group, which could point to a role in leukemia immune surveillance in these groups. These findings highlight the importance of enhancing (potentially leukemia-specific) NK and CIK cell functions in AML therapy, e.g., to stabilize remissions [73]. HMAs/VCLX and Kit-M might improve stability of remission and long-term survival, with further research into combination therapies potentially overcoming immune evasion needed [74].

ICM expression analysis showed, as observed in T cells, the highest frequencies of %NKKLRG1/NK (Figure 6d) in R_w/o_ (with lower levels in other groups, particularly in the patient treated with R_Kit-M_), indicating NK cell dysfunction [17]. Increased frequencies of CD107a^+^ and IFNγ^+^ NK and CIK cells (Figure 7a–d and Figure S7a–d) in CR/PR-patients and the Kit-M treated patient suggest preserved cytotoxic capacity despite checkpoint expression. Frequencies of NK279/NK followed a similar pattern, with the lowest levels in the Kit-M-treated patient (R_Kit-M_), what could potentially reflect a therapeutic effect [12]. Frequencies of NKTIM3/NK (Figure 6c) were elevated across all groups, with the highest frequencies in R_HMAs/VCLX_ and the patient treated with Kit-M (R_Kit-M_), which might indicate minimal impact of these therapies on frequencies of NKTIM3/NK. While TIM-3 is generally linked to immune suppression [13], it has also been associated with enhanced NK cytotoxicity and improved outcomes, warranting further investigation [16]. Elevated frequencies of 2B4- and TIGIT-positive NK cells were found in all groups (Figure 6e,f). This could point to a context-dependent role, with both inhibitory and activating functions reported [75,76]. Further research is required to clarify their precise effects.

### 4.4. Extended Immune Monitoring Strategies: (Potentially Leukemia-Specific) Immune Cell Detection and Their Role in Predicting Prognosis in AML Patients Post-HSCT

Precise immune monitoring in AML patients post-SCT is crucial for assessing immune reconstitution and predicting relapse risk. Traditional serological and functional assays lack the resolution to identify specific prognostic markers. Molecular techniques such as ‘quantitative PCR’ (qPCR) and ‘Real Time-PCR’ (RT-PCR) enable sensitive Minimal Residual Disease (MRD) detection but are limited to known genetic targets. Advanced methods like ‘digital PCR’ (dPCR) and ‘Next Generation Sequencing’ (NGS) enhance prognostic accuracy by detecting rare leukemic clones and providing comprehensive genomic profiling for risk stratification [77,78]. Multiparametric flow cytometry (MFC) is a powerful tool for prognostic immune monitoring. By characterizing immune cell subsets and (potentially) leukemia-specific responses, MFC identifies biomarkers/biomarker expressing cells predictive of relapse and long-term immune competence [79].

In our study, we used MFC to define immune signatures associated with post-SCT outcomes in AML patients: we grouped patients at relapse post-SCT into those who had died and those who had not in the ‘alive’ vs. the ‘deceased’ group. Kaplan–Meier analyses are generally regarded as the gold standard for survival comparisons. In our exploratory cohort, however, this approach was not ideally suited, as follow-up intervals were clinically driven and therefore irregular, the cohort size was small and heterogeneous, and some patients experienced early relapse-related deaths. We therefore applied a stratification into the outcome categories ‘alive’ vs. the ‘deceased’ group as described in Section 3.2. This strategy provided a clearer and more appropriate framework to compare immune profiles and enabled the detection of biologically plausible differences between groups, which may generate relevant hypotheses for future studies. The observed differences between long-term surviving and deceased patients should be interpreted cautiously, as the longer follow-up of survivors resulted in a greater contribution of samples obtained during treatment response or remission. Therefore, these immune profiles may reflect disease state and treatment-associated immune reconstitution in addition to any potential association with survival. A landmark analysis restricted to the first measurement at relapse would have comprised only 11 patients with incomplete data for several immune subsets and was therefore not considered sufficiently robust for the present exploratory analysis.

#### 4.4.1. Immune Monitoring of Myeloid Cells: Blasts and Dendritic Cells

At relapse after SCT frequencies of blasts were on average between 6 and 10% in the ‘alive’ and the ‘deceased’ group (Figure 4). Higher levels of blasts have been associated with poor outcome in various hematologic malignancies [80,81], potentially reflecting a dysregulated myeloid response that contributes to immune suppression and disease relapse. Such elevations in blast counts are typically linked to an immunosuppressive milieu [82]. However, in early relapse, these immune alterations may be less pronounced, which could explain why blast counts appear similarly elevated. Frequencies of DC/cells and DCleu/cells were also comparable in the two groups and were not predictive for survival in the patient cohorts studied at relapse.

#### 4.4.2. Immune Monitoring of Cells of the Adaptive Immune System

Analysis of adaptive immune cells revealed (significantly) elevated frequencies of various T cell subsets in the ‘alive’ group, highlighting their crucial role in long-term survival. Notably, increased frequences of T/cells and numerical trends in Tnn/cells, Tcm/cells, TIntβ7/cells and TCRγδ/cells (Figure 9a–e) confirm that T cell recovery and (antileukemic) diversity are associated with a favorable prognosis [27,83]. Potentially leukemia-specific (IFNγ-/CD107a-positive) T cells corroborated these findings (Figure 10). Although frequences of T_IFNγ_/cells (Figure 10a) were higher in the ‘alive’ group, other IFNγ expressing subsets did not show significant differences, suggesting that frequencies of IFNγ producing cells alone are not a decisive prognostic factor [84]. In contrast, CD107a expressing cells (reflecting potentially leukemia-specific/antileukemic cytotoxic potential), were significantly elevated or at least showed a numerical trend in multiple subsets (including memory and anti-tumor Intβ7- and TCRγδ-positive cells) in the ‘alive’ group (Figure 10b–f), underscoring the importance of functional cytotoxic T cell responses in controlling leukemic relapse and highlighting CD107a expressing cells as a valuable prognostic marker [37,85]. However, part of these differences may reflect the higher overall T cell frequency in survivors rather than increased cytotoxic activity on a per-cell basis.

Checkpoint marker analysis revealed increased frequencies of T2B4/T in the ‘alive’ group (Figure 9g) in survivors, potentially indicating functional activation rather than exhaustion in long-term survivors [25]. Nevertheless, without functional analyses, its specific role in our cohort cannot be determined. Conversely, slightly higher frequencies of T279/T (Figure 9f) in the ‘deceased’ group might suggest a more immunosuppressive environment with increased T cell dysfunction [19]. Other ICM-expressing cells displayed no significant differences and thus may not be useful as (only) prognostic markers.

#### 4.4.3. Immune Monitoring of Cells of the Innate Immune System

Innate immune cells, particularly CIK and NK cells, showed distinct patterns in the ‘alive’ compared to the ‘deceased’ group post-SCT. Frequencies of CIK/cells (Figure 11a) were significantly elevated in the ‘alive’ group, indicating their role in immune surveillance, while frequencies of NK/cells (Figure 11b) showed a similar numerical trend, highlighting the importance of both cell subsets in relapse prevention [86,87]. Potentially leukemia-specific immune responses, degranulating and particularly IFNγ-producing CIK and NK cells (Figure 11f–h), were significantly elevated in the ‘alive’ group, underscoring the relevance of functionally active, leukemia-specific innate immune cells in relapse prevention [88]. CD107a-expressing NK/CIK showed only moderate differences between the groups (except frequencies of CIK_107a_/cells, Figure 11h), suggesting that degranulation potential in innate cells alone may not be a robust prognostic indicator.

Frequencies of KLRG1- and CD279-expressing NK cells (Figure 11c,d) were higher in the ‘deceased’ group, indicating impaired cell function and poor prognosis as shown before [89,90], while frequencies of NK2B4/NK (Figure 11e) and other ICM-expressing cells showed no differences in the groups compared, thereby pointing to no significant role in antileukemic reactions and limiting their prognostic value.

Overall, these findings emphasize the functional role of innate immune cells, particularly CIK and NK cells, in influencing post-SCT outcomes in AML patients [74,86,87]. Their activity, particularly in the context of potentially leukemia-specific responses and checkpoint marker expression, may serve as valuable prognostic indicators. Further studies are needed to elucidate the mechanistic roles of these immune populations and explore their potential as therapeutic targets.

### 4.5. Leukemia-Specific T Cell Responses Mirror Therapeutic Milestones in Post-SCT Relapsed AML

Our Longitudinal profiling of CD107a^+^ T cells in three AML patients from the ‘alive’ cohort revealed temporally structured shifts in cytotoxic T cell activity corresponding to key therapeutic interventions. Patients from the ‘deceased’ cohort were excluded due to limited follow-up (≤4 time points), which made meaningful trajectory analysis unfeasible.

Frequencies of CD107a^+^ T cells (%T_107a_/T and %Tnn_107a_/T) rose notably during remission phases post-SCT and DLI. In patient 1650 (Figure 12a), a prominent post-DLI peak (71.5%) indicated strong T cell activation [24], followed by a rapid decline—suggesting only a transient immune engagement. However, due to the limited number of immune assessments performed shortly after DLI administration in our cohort, definitive conclusions regarding the immediate kinetics of DLI-induced responses remain elusive. This may reflect T cell exhaustion, reactivation of immunosuppressive pathways, regulatory T cell expansion, or immune checkpoint upregulation [91]. Patient 1654 (Figure 12b) showed an early decline during HMA/VCLX therapy with delayed immune recovery, while patient 1663 (Figure 12c) maintained elevated CD107a^+^ T cell levels during long-term remission post-third SCT, indicative of stable immune reconstitution. These findings are consistent with recent studies elucidating the mechanism of action of HMAs and VCLX, whereby HMAs may enhance antigen presentation, while VCLX sensitizes AML blasts via oxidative stress, enabling long-term remission [59,64]. The post-SCT period appears particularly permissive for reactivation and expansion of functional T cell populations. High frequencies of CD107a-expressing cells after SCT and DLI may suggest that these interventions may restore immunocompetence and facilitate the re-emergence of leukemia-reactive T cell clones. However, the rapid decline in patients 1650 and 1654 also highlights the challenges of maintaining these responses and underscores the role of immune regulatory circuits in restraining T cell efficacy over time [53]. Overall, these patient-specific immune trajectories underscore the importance of longitudinal immune monitoring and support combining SCT with immunomodulatory strategies to sustain anti-leukemic T cell responses in relapsed AML [54].

## 5. Limitations and Conclusions

Limitations of this study are: (1) CD107a surface expression identifies a (potentially leukemia-specific) degranulation/lysosomal-exocytosis-associated phenotype but does not prove direct cell cytotoxicity. (2) The correlation analyses were exploratory, included multiple comparisons, and were performed in a limited cohort without adjustment for multiple testing; causal conclusions therefore cannot be drawn. (3) Post-transplant clinical factors such as systemic immunosuppression and time from SCT to relapse were not systematically incorporated into the present analyses. Since these variables might affect immune reconstitution, they may have contributed to the observed differences between patient groups and should be considered in future prospective studies. (4) Our results obtained with a very heterogeneously treated patient cohort need validation in larger, independent cohorts. (5) Notably, due to the limited number of immune assessments conducted shortly after donor lymphocyte infusion (DLI), the immediate immunological effects of DLI could not be reliably assessed in this study and should be further investigated in future trials.

Despite these limitations, our findings emphasize the critical role of (potentially leukemia-specific) adaptive and innate immune responses in post-SCT survival, serving as valuable prognostic markers to predict patients’ outcomes at relapse after SCT (related or not related to treatment). Furthermore, our study emphasizes the critical role of both adaptive and innate immune responses in post-HSCT outcomes for relapsed AML patients. Elevated T cell cytotoxicity (e.g., CD107a expression) and enhanced leukemia-specific responses in CIK and NK cells were linked to better prognosis and relapse prevention. These findings suggest that boosting immune function, through strategies like immune checkpoint inhibitors and enhancing (leukemia-specific/DC mediated) T cell and NK cell activity, could improve patient outcomes in relapsed AML post-SCT, e.g., through improved GvL [5,92,93,94].

The frequencies of ICM (e.g., KLRG1, TIM-3, and TIGIT) expressing cells play a significant role in immune evasion, and their modulation could serve as important diagnostic and prognostic tools [31]. Advanced techniques like MFC could further refine immune monitoring and MRD detection, improving prognostic accuracy and treatment decisions [77].

The combination of hypomethylating agents with Venetoclax or Kit-M shows promise in restoring immune function [4], particularly Kit-M, by stimulating dendritic cells and boosting (leukemia-specific) T cell and NK cell responses. These treatments still require further refinement for long-term benefits. Moreover, immune checkpoint inhibitors targeting PD-1 and CTLA-4 could complement existing therapies by addressing immune escape mechanisms [65]. Especially the (immediate or later) immunological effects of DLI should be further investigated in future trials.

Given the complexity of AML and immune evasion, future therapies should focus on personalized medicine, tailoring treatments based on each patient’s immune profile. Integration of immune monitoring technologies will guide treatment decisions and optimize patient outcomes. To complement these findings, a parallel project in our group investigated immune signatures in AML patients prior to SCT [95]. Comparing pre- and post-transplant profiles is essential, as pre-transplant immune profiles might significantly influence post-SCT outcomes [96,97], guiding personalized treatment and refining prognostic models. Future studies should validate these markers in larger cohorts to refine post-SCT management and explore their therapeutic potential in relapse prevention.

## Figures and Tables

**Figure 1:**
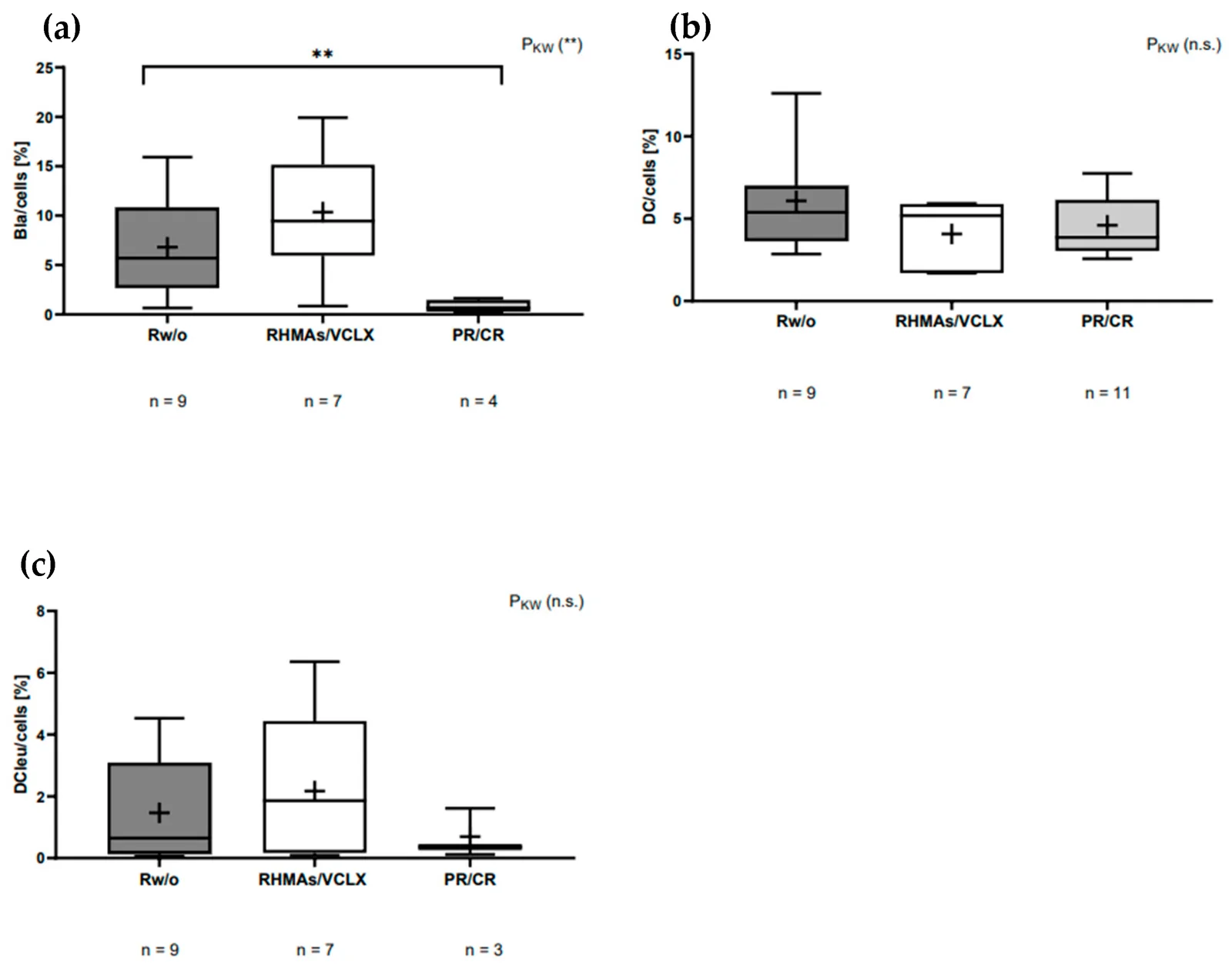
Frequencies of blasts (**a**) and DC subtypes (**b**,**c**) in patients’ blood in different stages of the disease and under different treatments. Given are median (—) and mean frequencies (+) ± standard deviation of blasts and DC subtypes in the groups (R_w/o_, R_HMAs/VCLX_, CR/PR). Explanations for treatment groups are given in Table 1; abbreviations for cell subtypes are given in Table 4. Statistical analyses were conducted using the non-parametric Kruskal–Wallis (P_KW_) test to compare all groups and the Mann–Whitney U test to compare two groups. Differences were considered as significantly different with *p*-values ≤ 0.05 (**) and non significantly different (n.s.) with *p*-values > 0.1.

**Figure 2:**
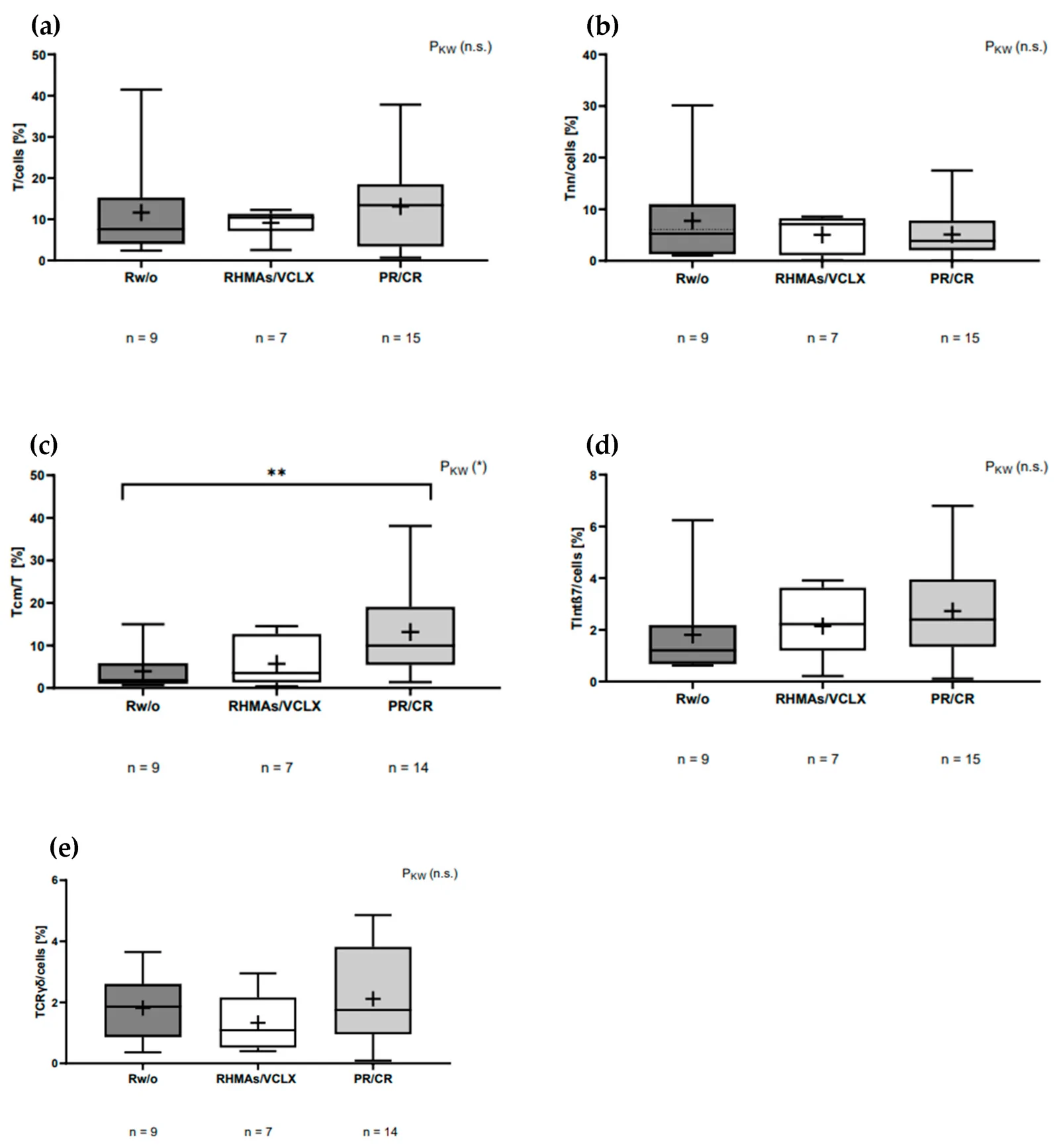
Frequencies of T cells (**a**) and T cell subtypes (Tnn (**b**), Tcm (**c**), TIntß7 (**d**), TCRγδ (**e**)) in patients’ blood in different stages of the disease and under different treatments. Given are median (—) and mean frequencies (+) ± standard deviation of T cells in the groups (R_w/o_, R_HMAs/VCLX_, CR/PR). Explanations for treatment groups are given in Table 1; abbreviations for cell subtypes are given in Table 4. Statistical analyses were conducted using the non-parametric Kruskal–Wallis (P_KW_) test to compare all groups and the Mann–Whitney U test to compare two groups. Differences were considered significantly different with *p*-values ≤ 0.05 (**) and a numerical trend with *p*-values ≤ 0.1 (*) and non significantly different (n.s.) with *p*-values > 0.1.

**Figure 3:**
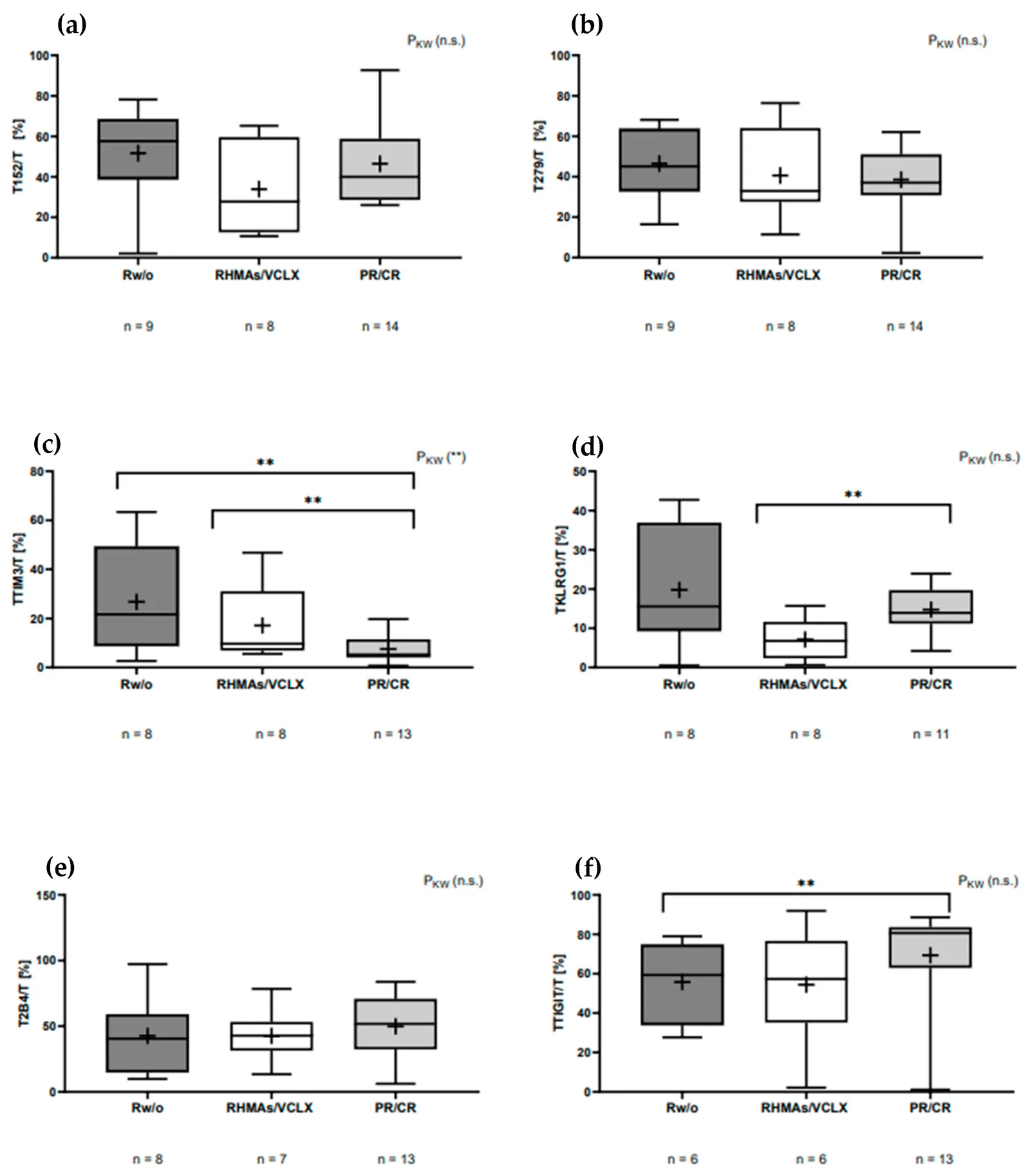
Frequencies of immune checkpoint-expressing T cells (T152 (**a**), T297 (**b**), TTim3 (**c**), TKLRG1 (**d**), T2B4 (**e**), TTIGIT (**f**)) in patients’ blood in different stages of the disease and under different treatments. Given are median (—) and mean frequencies (+) ± standard deviation of ICM-expressing innate immune cells in the groups (R_w/o_, R_HMAs/VCLX_, CR/PR). Explanations for treatment groups are given in Table 4, abbreviations for cell subtypes are given in Table 4. Statistical analyses were conducted using the non-parametric Kruskal–Wallis (P_KW_) test to compare all groups and the Mann–Whitney U test to compare two groups. Differences were considered significantly different with *p*-values ≤ 0.05 (**) and non significantly different (n.s.) with *p*-values > 0.1.

**Figure 4:**
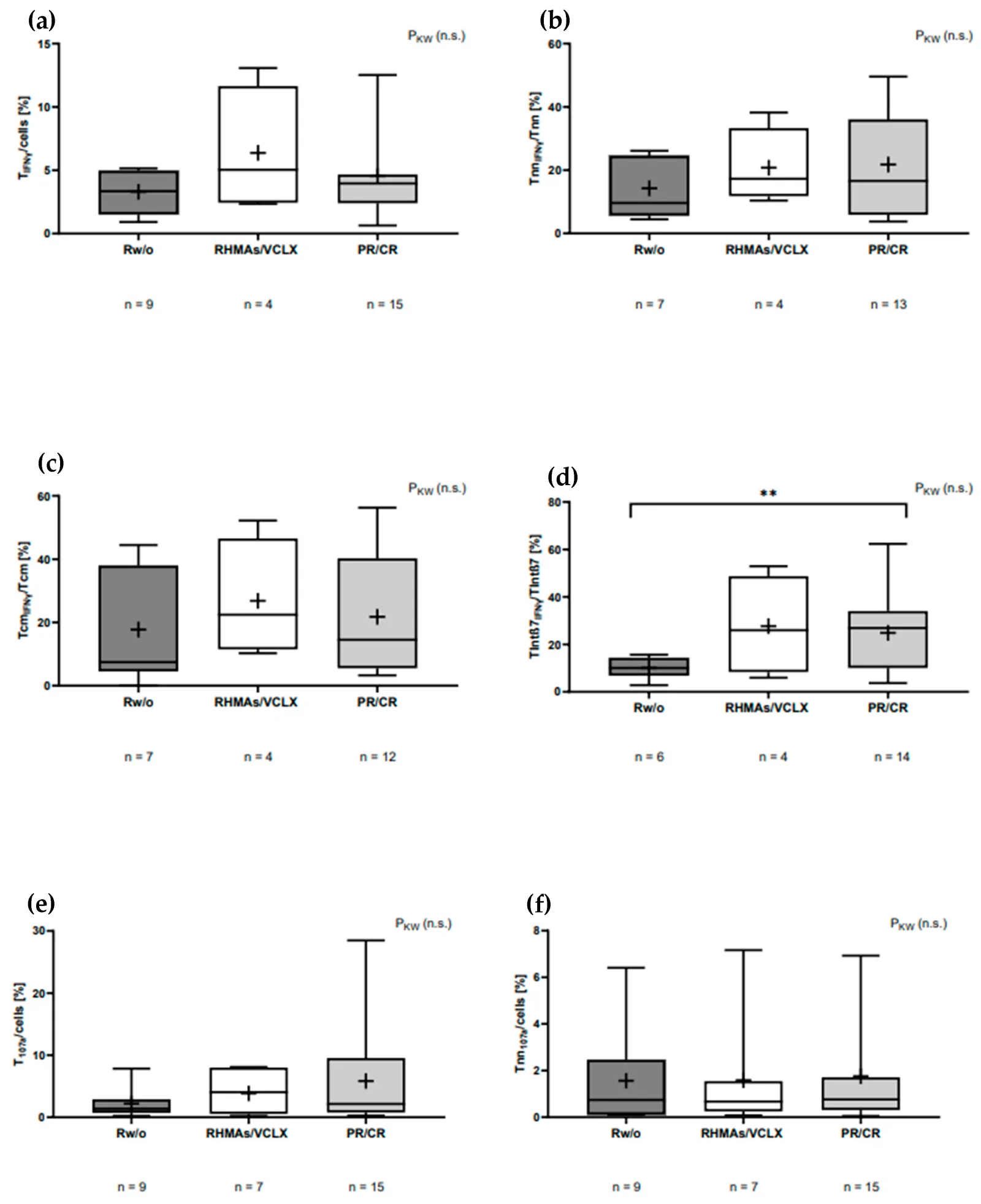

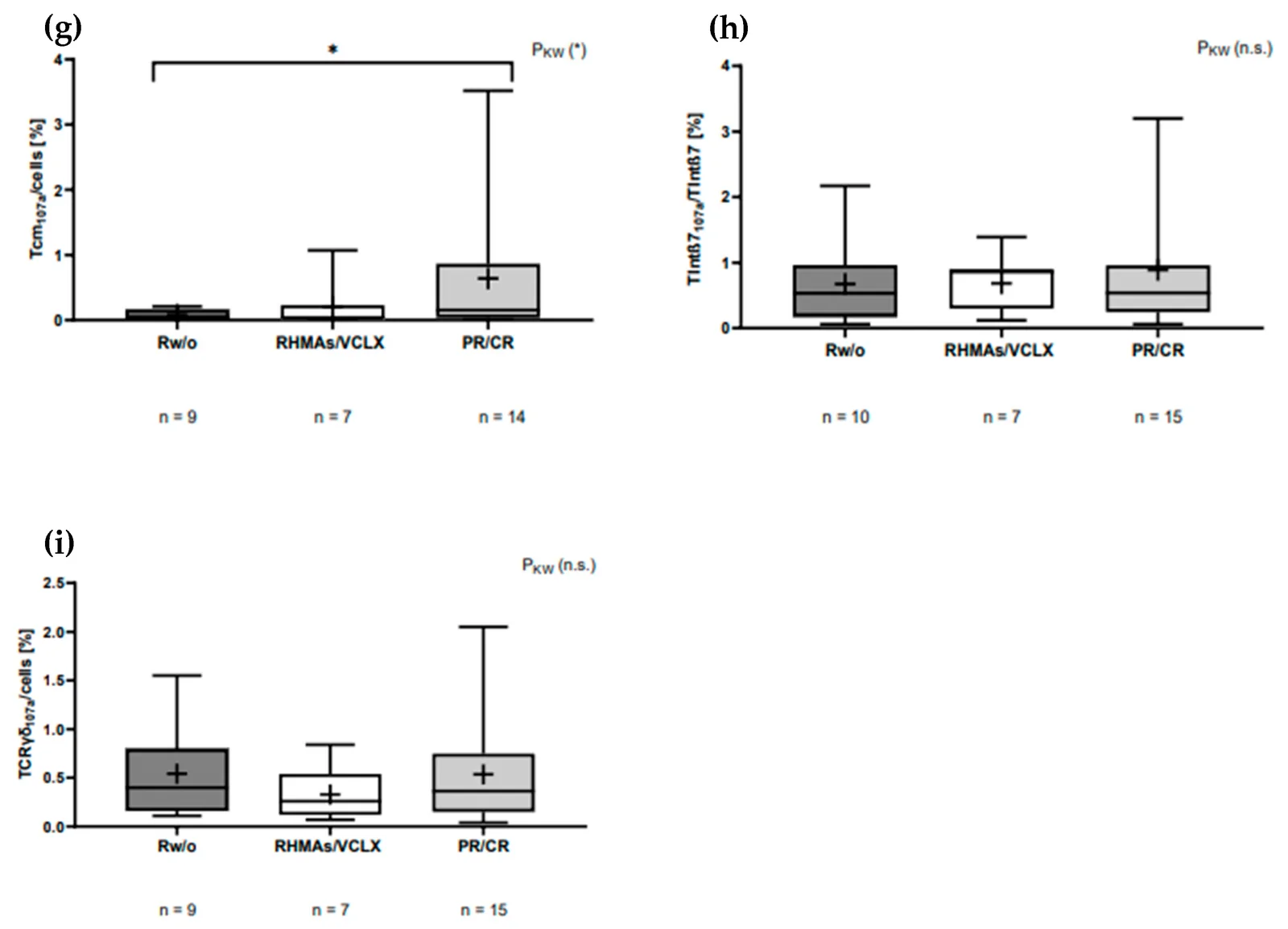
Frequencies of (potentially) leukemia-specific T cells (T_IFNγ_ (**a**), Tnn_IFNγ_ (**b**), Tcm_IFNγ_ (**c**), TIntβ7_IFNγ_ (**d**), T_107a_ (**e**), Tnn_107a_ (**f**), Tcm_107a_ (**g**), TIntβ7_107a_ (**h**), TCRγδ_107a_ (**i**)) in patients’ blood in different stages of the disease and under different treatments. Given are median (—) and mean frequencies (+) ± standard deviation of (potentially) leukemia-specific T cells in the groups (R_w/o_, R_HMAs/VCLX_, CR/PR). Explanations for treatment groups are given in Table 4, abbreviations for cell subtypes are given in Table 4. Statistical analyses were conducted using the non-parametric Kruskal–Wallis (P_KW_) test to compare all groups and the Mann–Whitney U test to compare two groups. Differences were considered significantly different with *p*-values ≤ 0.05 (**) and a numerical trend with *p*-values ≤ 0.1 (*) and non significantly different (n.s.) with *p*-values > 0.1.

**Figure 5:**
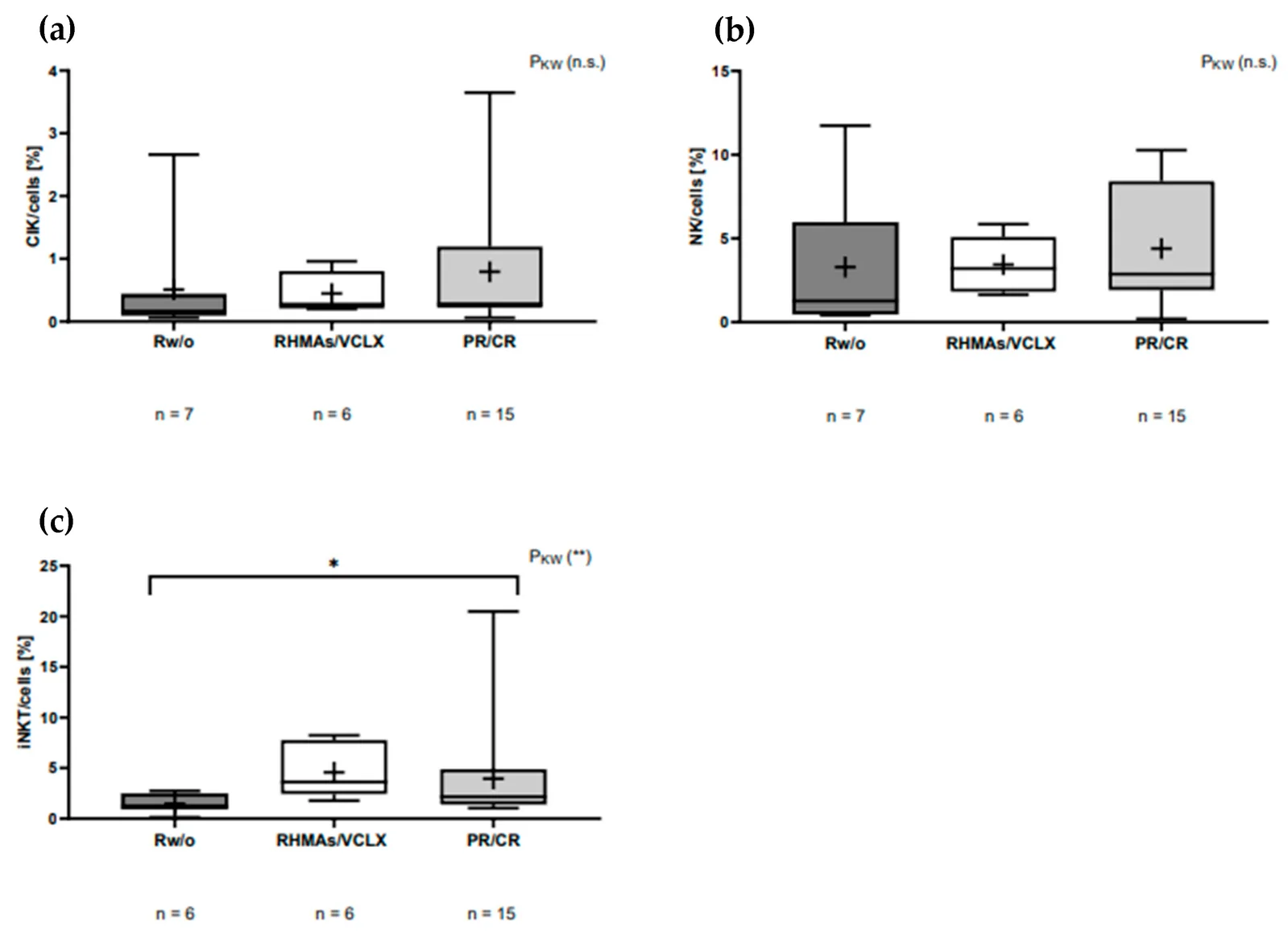
Frequencies of innate immune cells (CIK (**a**), NK (**b**), iNKT (**c**)) in patients’ blood in different stages of the disease and under different treatments. Given are median (—) and mean frequencies (+) ± standard deviation of innate immune cells in the groups (R_w/o_, R_HMAs/VCLX_, CR/PR). Explanations for treatment groups are given in Table 4; abbreviations for cell subtypes are given in Table 4. Statistical analyses were conducted using the non-parametric Kruskal–Wallis (P_KW_) test to compare all groups and the Mann–Whitney U test to compare two groups. Differences were considered significantly different with *p*-values ≤ 0.05 (**) and a numerical trend with *p*-values ≤ 0.1 (*) and non significantly different (n.s.) with *p*-values > 0.1.

**Figure 6:**
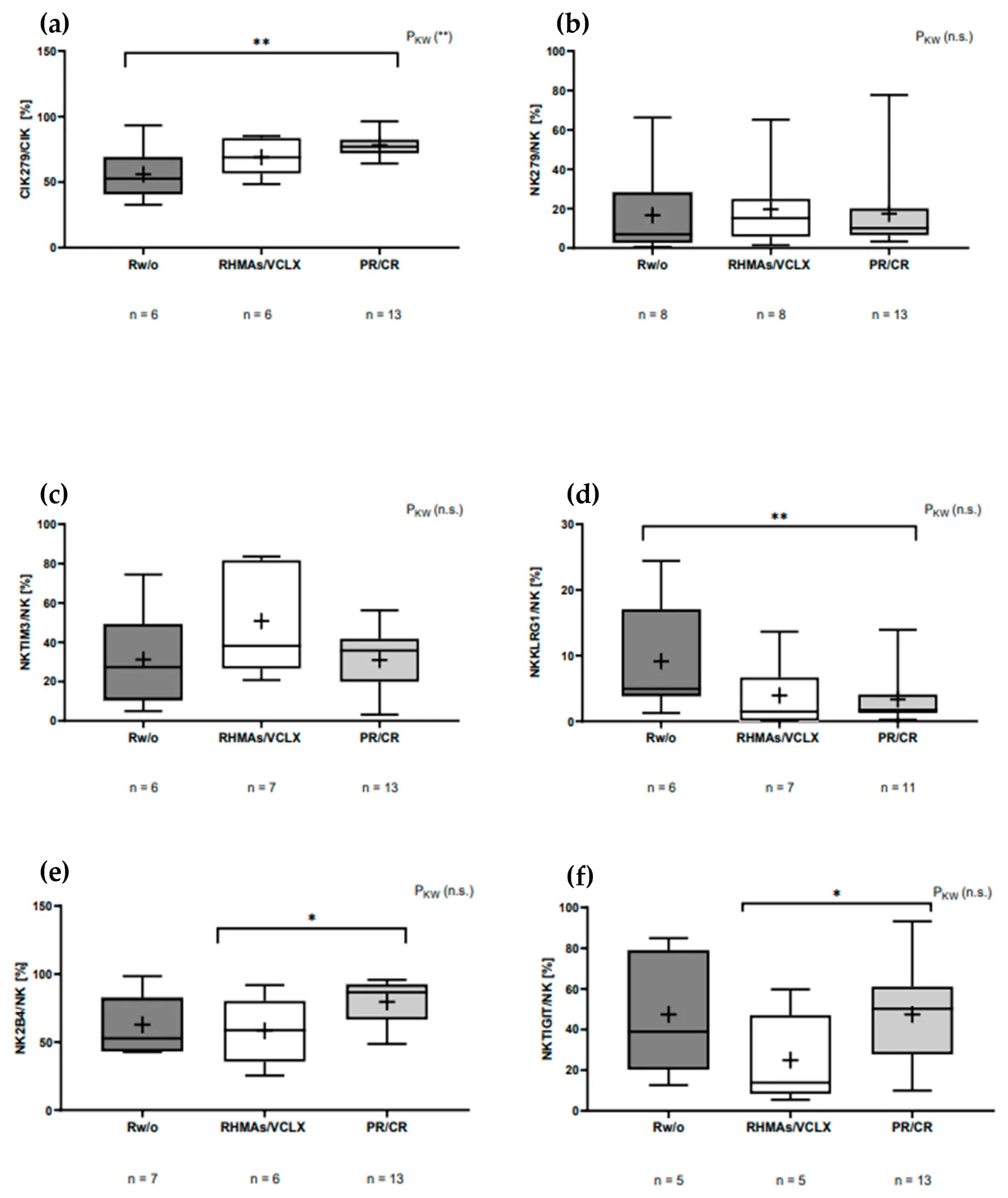
Frequencies of immune checkpointmarker-expressing innate immune cells (CIK279 (**a**), NK279 (**b**), NKTIM3 (**c**), NKKLRG1 (**d**), NK2B4 (**e**), NKTIGIT (**f**)) in patients’ blood in different stages of the disease and under different treatments. Given are median (—) and mean frequencies (+) ± standard deviation of ICM-expressing innate immune cells in the groups (R_w/o_, R_HMAs/VCLX_, CR/PR). Explanations for treatment groups are given in Table 4; abbreviations for cell subtypes are given in Table 4. Statistical analyses were conducted using the non-parametric Kruskal–Wallis (P_KW_) test to compare all groups and the Mann–Whitney U test to compare two groups. Differences were considered significantly different with *p*-values ≤ 0.05 (**) and a numerical trend with *p*-values ≤ 0.1 (*) and non significantly different (n.s.) with *p*-values > 0.1.

**Figure 7:**
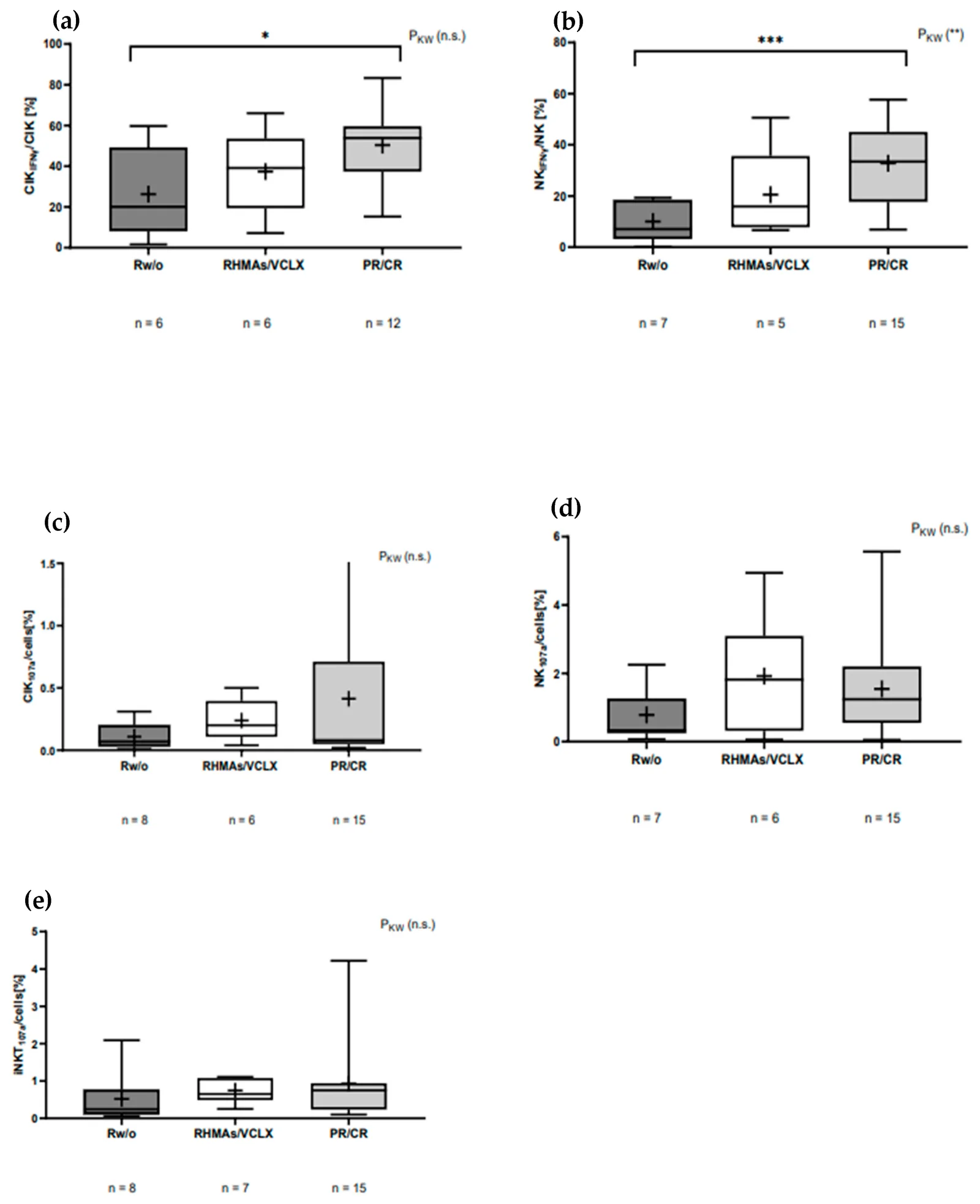
Frequencies of (potentially) leukemia-specific innate immune cells (CIK_IFNγ_ (**a**), NK_IFNγ_ (**b**), CIK_107a_ (**c**), NK_107a_ (**d**), iNKT_107a_ (**e**)) in patients’ blood in different stages of the disease and under different treatments. Given are median (—) and mean frequencies (+) ± standard deviation of (potentially) leukemia-specific innate immune cells in the groups (R_w/o_, R_HMAs/VCLX_, CR/PR). Explanations for treatment groups are given in Table 4; abbreviations for cell subtypes are given in Table 4. Statistical analyses were conducted using the non-parametric Kruskal–Wallis (P_KW_) test to compare all groups and the Mann–Whitney U test to compare two groups. Differences were considered highly significantly different with *p*-values ≤ 0.005 (***), significantly different with *p*-values ≤ 0.05 (**) and a numerical trend with *p*-values ≤ 0.1 (*) and non significantly different (n.s.) with *p*-values > 0.1.

**Figure 8:**
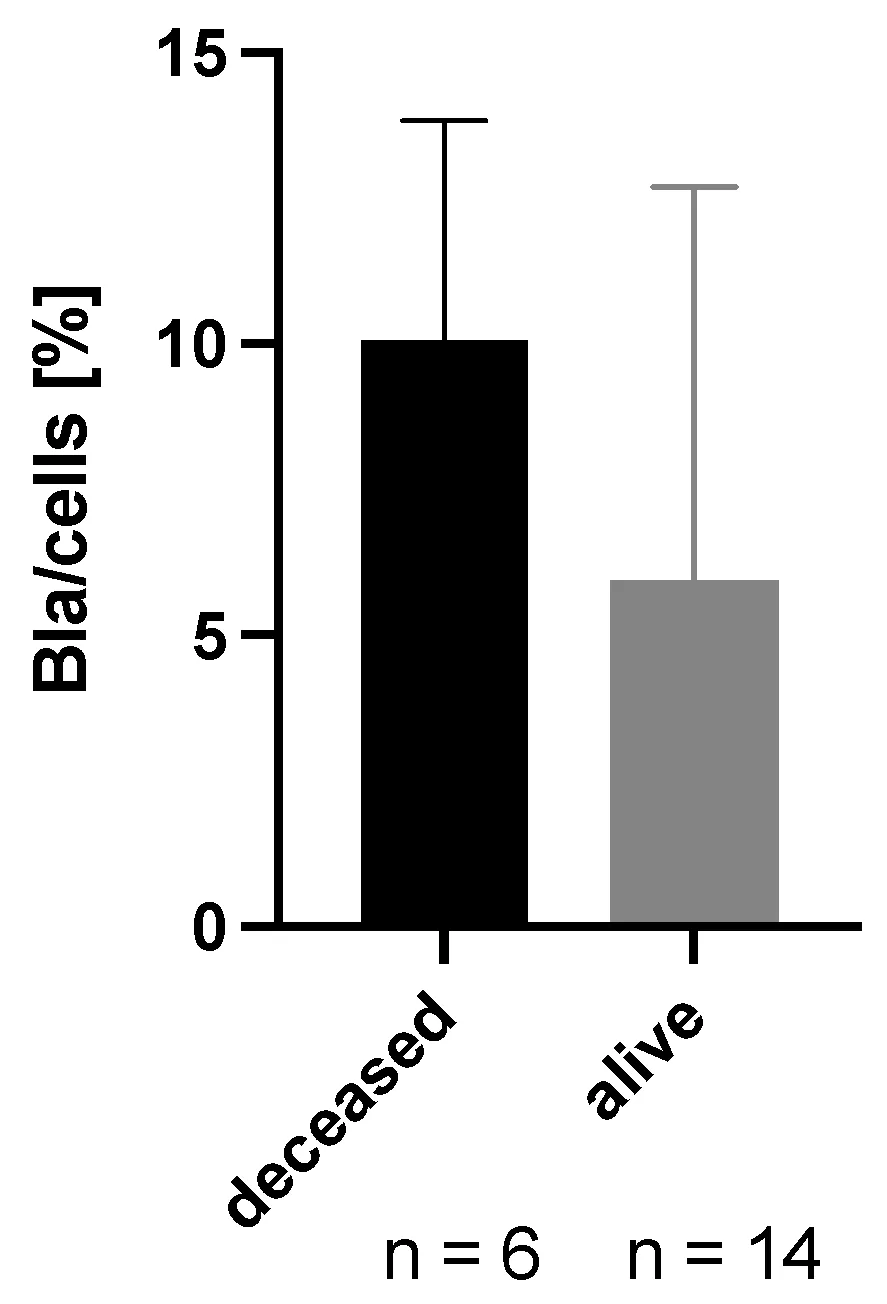
Frequencies of blasts in patients’ blood in deceased patients versus long-term survivors. Given are the mean frequencies (±standard deviation) of blasts in the ‘alive’ and the ‘deceased’ group of patients studied at relapse stage at the time of the 1st analysis. Explanations for patients’ status and stage at 1st analysis are given in Table 2; abbreviations for cell subtypes are given in Table 1. Statistical analyses were conducted using the Mann–Whitney U test to compare the two groups. Patients were classified into the two groups based on their survival status during the follow-up period: deceased (‘deceased’: n = 5) and long-term surviving (‘alive’: n = 6) AML patients. Only patients in the relapse stage at the time of the 1st analysis were included to ensure cohort homogeneity. Patient 1664, treated with a Kit M therapy, was excluded to avoid therapy-related bias. All individual measurements from the included patients were considered, without patient-wise pooling. The total number of measurements is represented by ‘n’.

**Figure 9:**
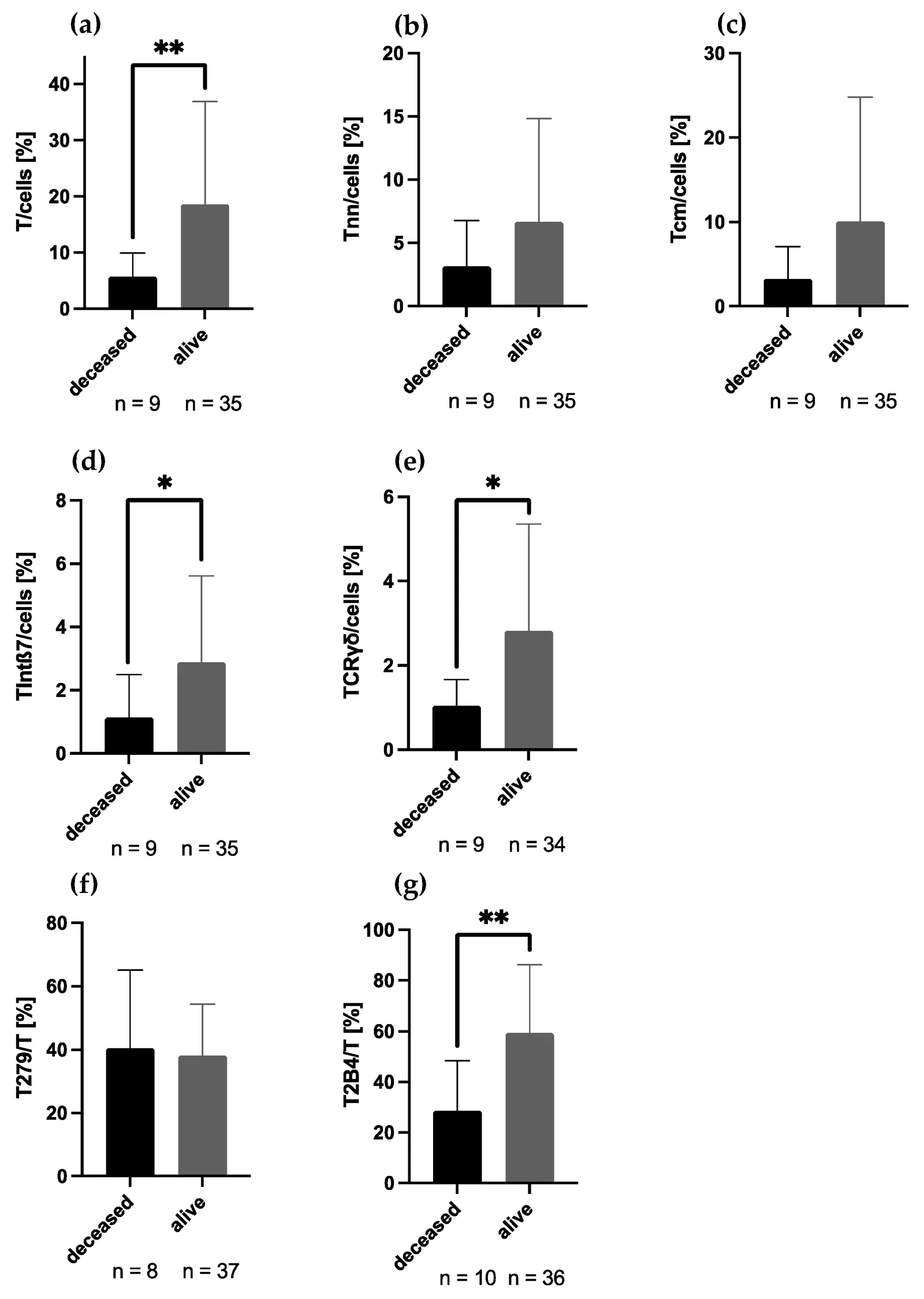
Frequencies of (immune checkpoint-expressing) T cells (**a**) in patients’ blood in deceased patients versus long-term survivors. Given are the mean frequencies (±standard deviation) of (ICM-expressing) T cells (Tnn (**b**), Tcm (**c**), TIntß (**d**), TCRγδ (**e**), T279 (**f**), T2B4 (**g**)) in the ‘alive’ and the ‘deceased’ group of patients studied at relapse stage at the time of the 1st analysis. Explanations for patients’ status and stage at 1st analysis are given in Table 2, abbreviations for cell subtypes are given in Table 1. Statistical analyses were conducted using the Mann–Whitney U test to compare the two groups. Patients were classified into the two groups based on their survival status during the follow-up period: deceased (‘deceased’: n = 5) and long-term surviving (‘alive’: n = 6) AML patients. Only patients in the relapse stage at the time of the 1st analysis were included to ensure cohort homogeneity. Patient 1664, treated with a Kit M therapy, was excluded to avoid therapy-related bias. All individual measurements from the included patients were considered, without patient-wise pooling. The total number of measurements is represented by ‘n’. Differences were considered significantly different with *p*-values ≤ 0.05 (**) and a numerical trend with *p*-values ≤ 0.1 (*).

**Figure 10:**
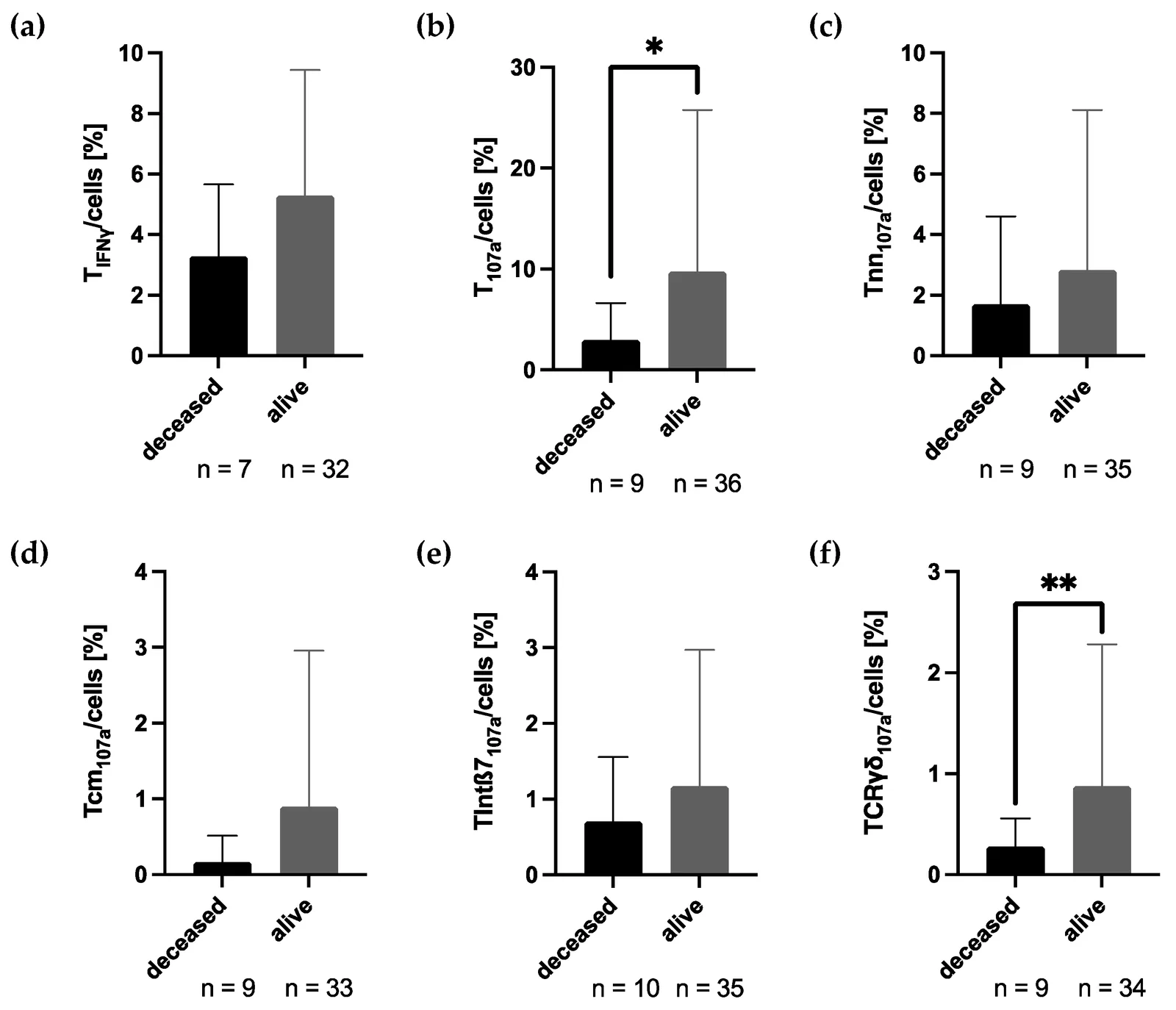
Frequencies of (potentially) leukemia-specific T cells in patients’ blood in deceased patients versus long-term survivors. Given are the mean frequencies (±standard deviation) of (potentially) leukemia-specific T cells (T_IFNγ_ (**a**), T_107a_ (**b**), Tnn_107a_ (**c**), Tcm_107a_ (**d**), TIntβ7_107a_ (**e**), TCRγδ (**f**)) in the ‘alive’ and the ‘deceased’ group of patients studied at relapse stage at the time of the 1st analysis. Explanations for patients’ status and stage at 1st analysis are given in Table 2, abbreviations for cell subtypes are given in Table 1. Statistical analyses were conducted using the Mann–Whitney U test to compare the two groups. Patients were classified into the two groups based on their survival status during the follow-up period: deceased (‘deceased’: n = 5) and long-term surviving (‘alive’: n = 6) AML patients. Only patients in the relapse stage at the time of the 1st analysis were included to ensure cohort homogeneity. Patient 1664, treated with a Kit M therapy, was excluded to avoid therapy-related bias. All individual measurements from the included patients were considered, without patient-wise pooling. The total number of measurements is represented by ‘n’. Differences were considered significantly different with *p*-values ≤ 0.05 (**) and a numerical trend with *p*-values ≤ 0.1 (*).

**Figure 11:**
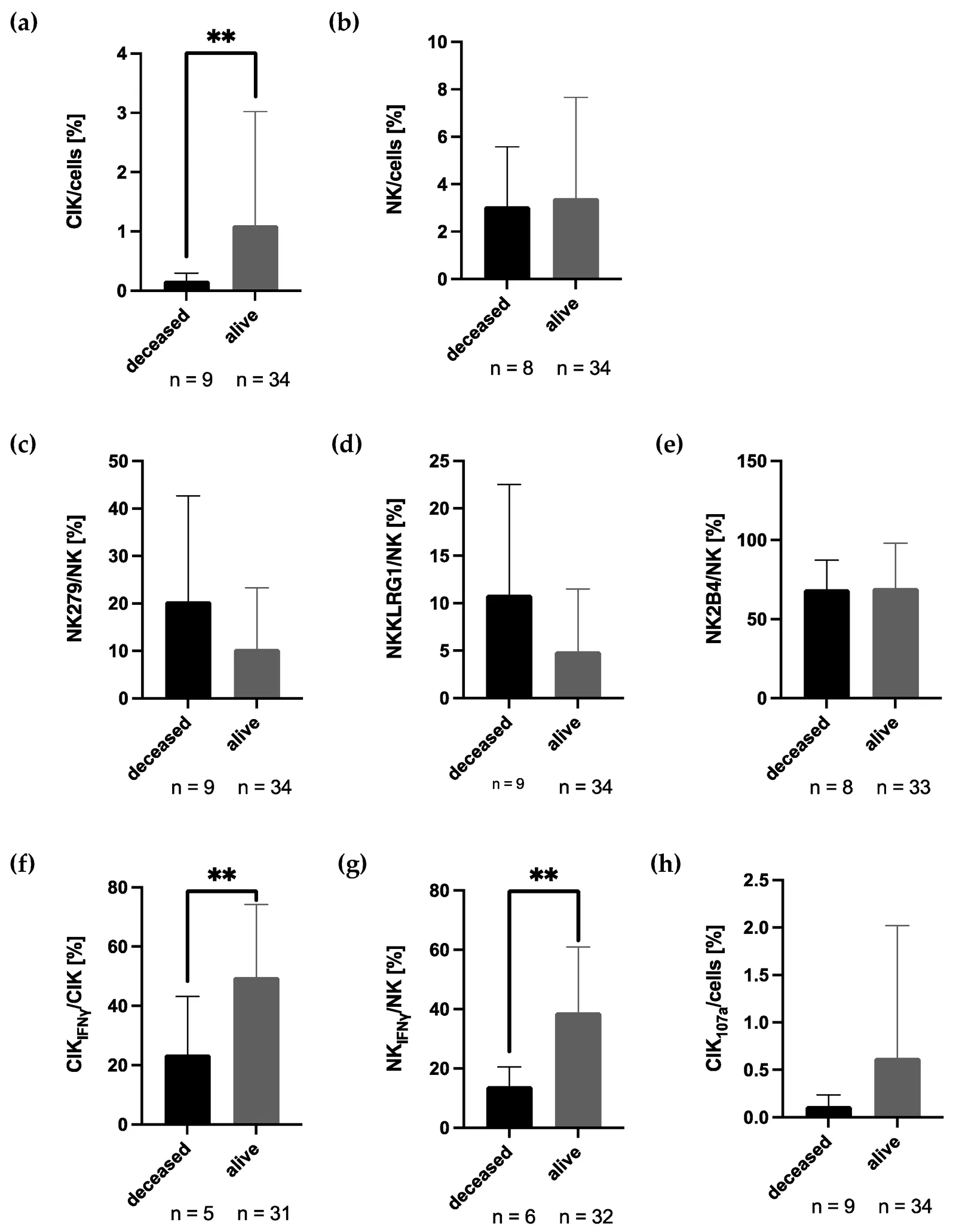
Frequencies of (leukemia-specific) innate immune cells in patients’ blood in deceased patients versus long-term survivors. Given are the mean frequencies (±standard deviation) of (potentially) leukemia-specific CIK (**a**) and NK cells (**b**) or their subsets (NK279 (**c**), NKKLRG1 (**d**), NK2B4 (**e**), CIK_IFNγ_ (**f**), NK_IFNγ_ (**g**), CIK_107a_ cells (**h**)) in the ‘alive’ and the ‘deceased’ group of patients studied at relapse stage at the time of the 1st analysis. Explanations for patients’ status and stage at 1st analysis are given in Table 2, abbreviations for cell subtypes are given in Table 1. Statistical analyses were conducted using the Mann–Whitney U test to compare the two groups. Patients were classified into the two groups based on their survival status during the follow-up period: deceased (‘deceased’: n = 5) and long-term surviving (‘alive’: n = 6) AML patients. Only patients in the relapse stage at the time of the 1st analysis were included to ensure cohort homogeneity. Patient 1664, treated with a Kit M therapy, was excluded to avoid therapy-related bias. All individual measurements from the included patients were considered, without patient-wise pooling. The total number of measurements is represented by ‘n’. Differences were considered significantly different with *p*-values ≤ 0.05 (**).

**Figure 12:**
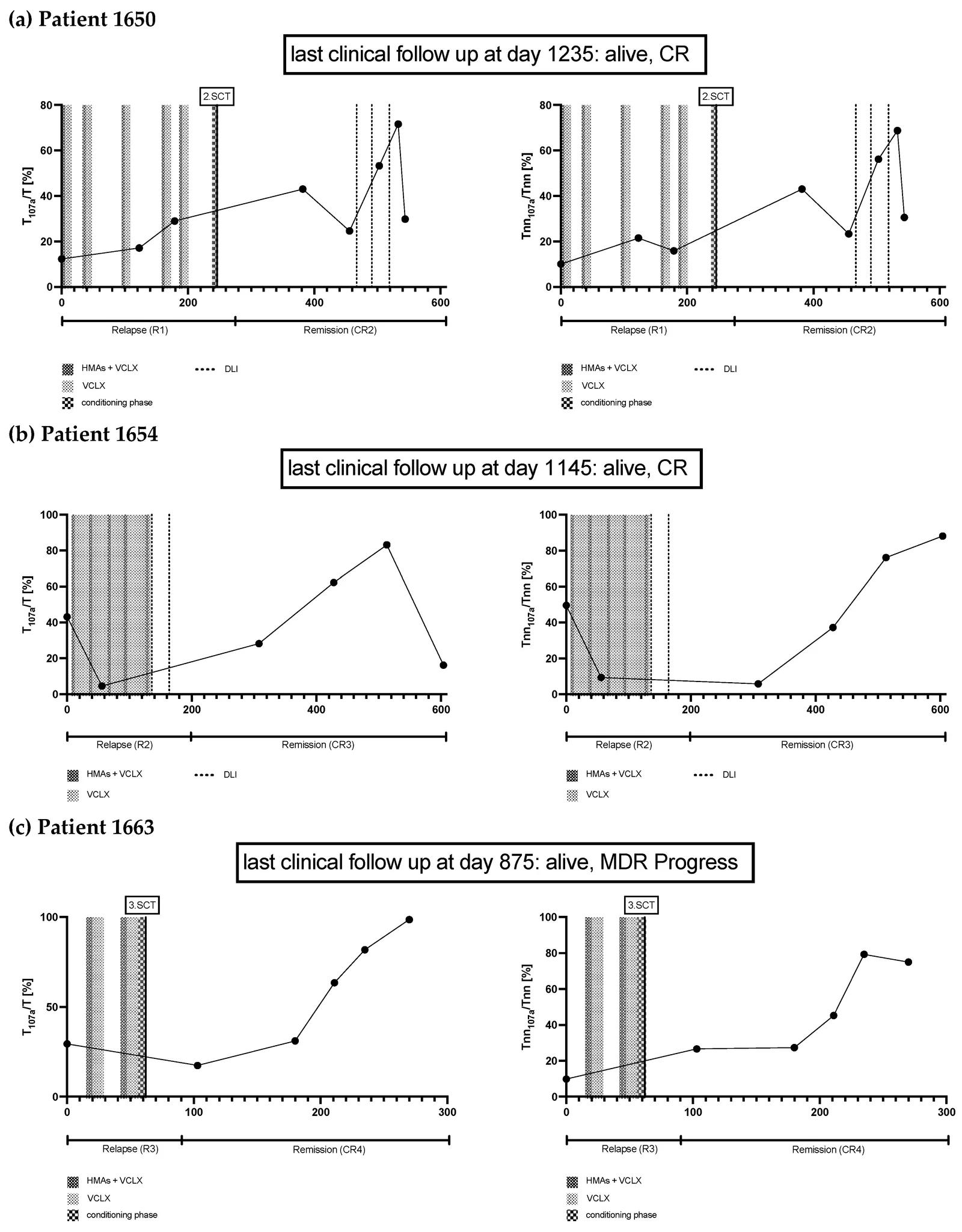
Follow-up analysis of (potentially) leukemia-specific adaptive immune cells in the course of disease and treatment. Given are time-course analysis of (potentially) leukemia-specific adaptive immune cells in the course of the disease and treatment in patients 1650 (**a**), 1654 (**b**) and 1663 (**c**). Legend: x-axis: days since initiation of follow-up analysis, y-axis: frequencies of cells, HMAs + VCLX: treatment with hypomethylating agents (Decitabine or Vidaza) in combination with Venetoclax, VCLX: treatment with only Venetoclax, DLI: donor lymphocyte infusion, 2.SCT: second stem cell transplantation since diagnosis, 3.SCT: third stem cell transplantation since diagnosis.

**Table 1:** Cell subtypes.

Cell Type	Name of Subgroups	Surface Marker	Abbreviation	Reference
Myeloid cells (referred to as non-lymphoid cells * and their subsets)				
Blast cells	Leukemic blasts	Bla^+^ (CD34^+^, CD117^+^)	Bla/cells *	[10]
Dendritic cells	Dendritic cells	DC^+^ (CD80^+^, CD206^+^)	DC/cells *	[10]
	Leukemia-derived DC	DC^+^Bla^+^	DCleu/cells *	[10]
			DCleu/Bla	[10]
			DCleu/DC	[10]
***Immune reactive cells (referred to as lymphoid cells # and their subsets)***				
Innate immune system	NK cells	CD3^−^CD56^+^	NK/cells #	[11]
	CTLA4-expressing NK cells	CD3^−^CD56^+^CD152^+^	NK152/NK and cells #	[12]
	PD1-expressing NK cells	CD3^−^CD56^+^CD279^+^	NK279/NK and cells #	[13]
	2B4-expressing NK cells	CD3^−^CD56^+^CD2B4^+^	NK2B4/NK and cells #	[14]
	TIGIT-expressing NK cells	CD3^−^CD56^+^TIGIT^+^	NKTIGIT/NK and cells #	[15]
	TIM3-expressing NK cells	CD3^−^CD56^+^TIM3^+^	NKTIM3/NK and cells #	[16]
	KLRG1-expressing NK cells	CD3^−^CD56^+^KLRG1^+^	NKKLRG1/NK and cells #	[17]
	CIK cells	CD3^+^CD56^+^	CIK/cells #	[11]
	PD-1-expressing CIK cells	CD3^+^CD56^+^CD279^+^	CIK279/CIK and cells #	[18,19]
	Invariant natural killer T cells	6b11^+^	iNKT/cells #	[18]
Adaptive immune system	CD3^+^ pan T cells	CD3^+^	T/cells #	[20]
	Non-naive T cells	CD3^+^CD45RO^+^	Tnn/T and cells #	[21]
	Central memory T cells	CD3^+^CD45RO^+^CD197^+^	Tcm/T and cells #	[21]
	TCRγδ T cells	CD3^+^TCRγδ^+^	TCRγδ/T and cells #	[22]
	Integrine β7 T cells	CD3^+^Intβ7^+^	TIntβ7/T and cells #	[22]
	CTLA4-expressing T cells	CD3^+^CD152^+^	T152/T and cells #	[23]
	PD1-expressing T cells	CD3^+^CD279^+^	T279/T and cells #	[24]
	2B4-expressing T cells	CD3^+^CD2B4^+^	T2B4/T and cells #	[25]
	TIGIT-expressing T cells	CD3^+^TIGIT^+^	TTIGIT/T and cells #	[15]
	TIM3-expressing T cells	CD3^+^TIM3^+^	TTIM3/T and cells #	[26]
	KLRG1-expressing T cells	CD3^+^KLRG1^+^	TKLRG1/T and cells #	[17]
***Intracellularly IFNγ-producing cells***				
Innate immune cells	NK cells	IFNγ^+^CD3^−^CD56^+^	NK_IFNγ_/NK and cells #	[10]
	CIK cells	IFNγ^+^CD3^+^CD56^+^	CIK_IFNγ_/CIK and cells #	[10]
Adaptive immune system	CD3^+^ pan T cells	IFNγ^+^CD3^+^	T_IFNγ_/T and cells #	[10]
	Non-naive T cells	IFNγ^+^CD3^+^CD45RO^+^	Tnn_IFNγ_/Tnn and cells #	[10]
	Central memory T cells	IFNγ^+^CD3^+^CD45RO^+^CD197^+^	Tcm_IFNγ_/Tcm and cells #	[10]
	Integrine β7 T cells	IFNγ^+^CD3^+^Intβ7^+^	TIntβ7_IFNγ_/TIntβ7 and cells #	[22]
***Degranulating cells (CD107a)***				
Innate immune cells	NK cells	CD107a^+^CD3^−^CD56^+^	NK_107a_/NK and cells #	[10]
	CIK cells	CD107a^+^CD3^+^CD56^+^	CIK_107a_/CIK and cells #	[10]
	Invariant natural killer T cells	CD107a^+^6b11^+^	iNKT_107a_/iNKT and cells #	[10]
Adaptive immune system	CD3^+^ pan T cells	CD107a^+^CD3^+^	T_107a_/T and cells #	[10]
	Non-naive T cells	CD107a^+^CD3^+^CD45RO^+^	Tnn_107a_/Tnn and cells #	[10]
	Central memory T cells	CD107a^+^CD3^+^CD45RO^+^CD197^+^	Tcm_107a_/Tcm and cells #	[10]
	TCRγδ T cells	CD107a^+^CD3^+^ TCRγδ^+^	TCRγδ_107a_/TCRγδ and cells #	[27]
	Integrine β7 T cells	CD107a^+^CD3^+^Intβ7^+^	TIntβ7_107a_/TIntβ7 and cells #	[22]

* Refered to a ‘Non-Lymphocyte’ gate; # refered to a ‘lymphocyte’ gate.

**Table 2:** Patients’ characteristics.

Patient Number	Sex	Age at 1st Analysis	WHO Classification	Stage at 1st Analysis	Chimerism at 1st Analysis in BM Total/T Cell	Blast Count at 1st Analysis in BM/PB	Last Day of Follow-Up After 1st Analysis/Status	Blast Phenotype at Diagnosis	Number of SCT	Number of Measurements in the Course of Observation	Analyzed in Following Treatment Groups
1632	f	56	AML with defining genetic abnormalities	relapse	35%/88%	72%/75%	52/deceased	34, 117, 33, 13, 7	2	1	R_w/o_
1641	f	64	AML, post-cytotoxic therapy	relapse	62%/46%	44%/5%	1360/alive	34, 33, 13, 64, 14, 65, 117, 15	2	5	R_w/o_, R_HMAs/VCLX_, CR/PR
1650	f	64	AML, myelodysplasia-related	relapse	86%/99%	13%/1%	1235/alive	34, 13, 117, 56	2	9	R_w/o_, R_HMAs/VCLX_, CR/PR
1654	m	71	AML with defining genetic abnormalities	relapse	97% */100% *	Punctio sicca/5%	1145/alive	13, 34, 117	1	6	R_w/o_, R_HMAs/VCLX_, CR/PR
1655	m	66	AML, myelodysplasia-related	relapse	16%/86%	90%/6%	1136/alive	34, 13, 117,	2	6	R_HMAs/VCLX_, CR/PR
1656	f	42	AML with defining genetic abnormalities	relapse	86%/94%	23%/11%	330/deceased	34, 13, 33, 117, 56	2	3	R_w/o_, R_HMAs/VCLX_
1658	f	59	AML defined by differentiation	relapse	75%/82%	72%/14%	10/deceased	33, 64, 14, 56	1	1	R_w/o_
1660	m	70	MDS defined by morphology	relapse	81%/99%	8%/6%	286/deceased	34, 117, 33, 13, 123	2	4	R_w/o_, R_HMAs/VCLX_, CR/PR
1663	f	46	AML defined by differentiation	relapse	6%/99%	80%/10%	875/alive	34, 117, 33, 7, 13, 65, 15	3	6	R_w/o_, CR/PR
1664	m	65	AML with defining genetic abnormalities	relapse	89% */100% *	Punctio sicca/3%	265/deceased	34, 117, 33, 13, 65	2	22	R_HMAs/VCLX_, R_Kit-M_
1665	m	68	AML, myelodysplasia-related	relapse	9%/81%	5%/2%	784/alive	33, 34, 13, 15	1	6	R_w/o_, CR/PR
1674	f	51	AML, myelodysplasia-related	relapse	82%/100%	21%/7%	69/deceased	34, 13, 33, 117	1	2	R_w/o_, R_HMAs/VCLX_
1640	f	73	AML with defining genetic abnormalities	partial remission	94%/88%	4%/1%	1272/deceased	33, 7, 117, 13, 34	2	12	CR/PR
1603	f	32	AML with recurrent genetic aberrations	complete remission	/	/	771/alive	15, 33, 34, 117	1	8	CR/PR

Legend: f—female, m—male, AML—acute myeloid leukemia, WHO—World Health Organization, Last day of follow up—refers to the final date on which clinical information was available after 1st analysis, SCT—stem cell transplantation, Number of SCT—total number of SCTs since diagnosis, R_w/o_—relapse without therapy, R_HMAs/VCLX_—relapse under/after hypomethylating agents and/or Venetoclax treatment, R_Kit-M_—relapse under/after Kit M treatment, PR—Partial Remission, CR—Complete Remission under/after different treatment regimes, PB—peripheral blood, BM—bone marrow, * measured in peripheral blood lymphocytes.

**Table 3:** Patients’ individually received therapies during follow-up.

Patients	Individually Received Therapies
**1603**	**d0: CR1** d0–59: Cyclosporin A with Mycophenolate last measurement: d604
**1632** (only 1st analysis)	**d0: R2** **d52: death** last measurement: d0
**1640**	**d0: PR1** d6–10: Vidaza/Venetoclax, d11–32: Venetoclax, d36–40: Vidaza/Venetoclax, d41–56: Venetoclax, d71–75: Vidaza/Venetoclax, d76–91: Venetoclax, d113–120: conditioning followed by SCT, **d147: CR2** d303, d345: DLI, **d364: R2** d370–374: Vidaza/Venetoclax, d375–383: Venetoclax, **d407: PR2** d418–427: Vidaza/Venetoclax, d428–440: Venetoclax, d441–445: Vidaza, d446–447: Vidaza/Venetoclax, d448–461: Venetoclax, d469–473: Vidaza/Venetoclax, d474–483: Venetoclax, **d505: CR3** d513–d675: 9xNivolumab, **d681: R3** d699–703: Vidaza/Venetoclax, d704–705: Venetoclax, d727–731: Vidaza/Venetoclax, d732–733: Venetoclax, d756–758: Vidaza/Venetoclax, d759–763: Decitabine/Venetoclax, d764–772: Venetoclax, d805–809: Decitabine, d842–846: Decitabine **d869: PR3** **d1272: death** last measurement: d869
**1641**	**d0: R1** d1–5: Azacitidine/Venetoclax, d6–12: Venetoclax, d40–46: Azacitidine/Venetoclax, d47–49: Venetoclax, d104–120: conditioning followed by SCT **d148: CR2** last measurement: d818
**1650**	**d0: R1** d1–5: Vidaza/Venetoclax, d6–12: Venetoclax, d33–37: Vidaza/Venetoclax, d38–46: Venetoclax, d95–99: Vidaza/Venetoclax, d100–108: Venetoclax, d158–162: Vidaza/Venetoclax, d163–171: Venetoclax, d186–190: Vidaza/Venetoclax, d191–199: Venetoclax, d238–246: conditioning followed by SCT **d274: CR2** d467, d491, d519: DLI last measurement: d544
**1654**	**d0: R2** d7–11: Vidaza/Venetoclax, d12–34: Venetoclax, d35–39: Vidaza/Venetoclax, d40–64: Venetoclax, d65–69: Vidaza/Venetoclax, d70–90: Venetoclax, d91–95: Vidaza/Venetoclax, d96–125: Venetoclax, d126–130: Vidaza/Venetoclax, d131–132: Venetoclax, d136, d164: DLI **d199: CR3** last measurement: d604
**1655**	**d0: R1** d0–4: Decitabine/Venetoclax, d5–22: Venetoclax, d27–36: Decitabine/Venetoclax, d37-d40: Venetoclax, d50: Cytarabin/Daunorubicin, d51–54: Cytarabin, d57–70: conditioning followed by SCT **d98: CR2** d213, d241: DLI last measurement: 434
**1656**	**d0: R1** d0-d1: Venetoclax, d2–11: Decitabine/Venetoclax, d12–14: Venetoclax, d44–47: Cytarabin, d49–55: conditioning followed by SCT **d85: CR2** **d143: R2** **d330: death** last measurement: d176
**1658** (only 1st analysis)	**d0: R1** **d10: death** last measurement: d0
**1660**	**d0: R1** d7–11: Vidaza/Venetoclax, d12–20: Venetoclax, d40–44: Vidaza/Venetoclax, d45–51: Venetoclax, d67–71: Vidaza/Venetoclax, d72–86: Venetoclax, d96–100: Vidaza/Venetoclax, d101–110: Venetoclax, d130–134: Vidaza/Venetoclax, d135–143: Venetoclax, d229–237: conditioning followed by SCT **d263: CR2** **d286: death** last measurement: d263
**1663**	**d0: R3** d15–19: Decitabine/Venetoclax, d20–28: Venetoclax, d42–46: Decitabine/Venetoclax, d47–55: Venetoclax, d56–62: conditioning followed by SCT **d90: CR4** last measurement: d270
**1664**	**d0: R2** d0–4, d28–32, d64–68, d99–103: Vidaza, d120–124, d127–131, d136–140, d143–147, d172–176, d194–198, d210–214, d224–228: Kit-M **d265: Death**
**1665**	d0: R1 d19–25: Vidaza/Venetoclax, d47–53: Vidaza/Venetoclax, d54–74: Venetoclax, d75–81: Vidaza/Venetoclax, d89–94: Venetoclax **d95: CR2** d95–104: Venetoclax, d105–111: Vidaza/Venetoclax, d112–142: Venetoclax, d143–149: Vidaza/Venetoclax, d150–170: Venetoclax, d171–177: Venetoclax, d178–194: Venetoclax, d199–205: Vidaza last measurement: d215
**1674**	**d0: R1** d6–10: Decitabine **d69: death** last measurement: d27

**Table 4:** Composition of treatment groups in patients at relapse after SCT.

Name of Treatment Groups	Abbreviation	Composition of Treatment Groups
Relapse without therapy	R_w/o_	(n = 10)
Relapse under/after hypomethylating agents and/or Venetoclax	R_HMAs/VCLX_	- hypomethylating agents and venetoclax (n = 5) - hypomethylating agents (n = 2) - Venetoclax (n = 1)
Relapse under/after Kit-M	R_Kit-M_	(n = 1)
Partial remission and Complete remission under/after different treatment regimens	PR/CR	- PR: without therapy (n = 1) - PR: hypomethylating agents and Venetoclax (n = 1) - PR: hypomethylating agents and Venetoclax and donor lymphocytes infusion (n = 1) - CR: without therapy (n = 1) - CR: Cyclosporin A with Mycophenolat (n = 1) - CR: second/third stem cell transplantation (n = 6) - CR: second stem cell transplantation and donor lymphocyte infusion (n = 1) - CR: hypomethylating agents and Venetoclax (n = 2) - CR: Nivolumab (n = 1)

Legend: n: number of patients who have received the respective therapy in the course of disease and treatment.

## Data Availability

Data are provided within the manuscript or Supplementary Information files.

## Supplementary Materials

The following supporting information can be downloaded at: https://www.mdpi.com/article/10.3390/cancers18213421/s1, Table S1: Treatment protocol of Kit-M (GM-CSF + PGE1) for patient 1664; Table S2: Antibody panels used for cell subtype quantification; Figure S1: Frequencies of blasts and DC subtypes in patients’ blood in different stages of the disease and under different treatments; Figure S2: Frequencies of T cells and T-cell subtypes in patients’ blood in different stages of the disease and under different treatments; Figure S3: Frequencies of immune checkpoint-expressing T cells in patients’ blood in different stages of the disease and under different treatments; Figure S4: Frequencies of (potentially) leukemia-specific T cells in patients’ blood in different stages of the disease and under different treatments; Figure S5: Frequencies of innate immune cells in patients’ blood in different stages of the disease and under different treatments; Figure S6: Frequencies of immune checkpoint marker-expressing innate immune cells in patients’ blood in different stages of the disease and under different treatments; Figure S7: Frequencies of leukemia-specific innate immune cells in patients’ blood in different stages of the disease and under different treatments; Figure S8: Gating strategy of (potentially) leukemia-specific immune cells using flow cytometry and analysis of immune cell subsets.
