# Supplementary material for: Monitoring of (Potentially Leukemia-Specific) Immune Cells After AML Relapse Post-Transplantation Across Treatment Groups and Disease Stages
Source: Cancers (Basel). 2026 Oct 22;18(21):3421. doi: 10.3390/cancers18213421 (PMC13761525; doi:10.3390/cancers18213421)
Supplement: Supplementary file 1 [file cancers-18-03421-s001.zip › cancers-4556236-supplementary.pdf]

Supplementary Material:

Table S1: Treatment protocol of Kit M (GM-CSF + PGE1) for patient 1664

Phase	Day	Drug	Dosage (i.v.)	Schedule
Ramp up phase	120-122	GM-CSF<br>PGE1	75 µg/m²<br>20 µg	4 hours (9 am - 1 pm)<br>2 hours (1 pm - 3 pm)
	123-124	GM-CSF<br>PGE1	75 µg/m²<br>40 µg	4 hours (9 am - 1 pm)<br>2 hours (1 pm - 3 pm)
	127-131	GM-CSF<br>PGE1	75 µg/m²<br>40 µg	4 hours (9 am - 1 pm)<br>2 hours (1 pm - 3 pm)
	136-140	GM-CSF<br>PGE1	75 µg/m²<br>40 µg	4 hours (9 am - 1 pm)<br>2 hours (1 pm - 3 pm)
	143-147	PGE1<br>GM-CSF<br>PGE1	40 µg<br>75 µg/m²<br>40 µg	2 hours (9 am - 11 am)<br>4 hours (11 am - 3 pm)<br>2 hours (3 pm - 5 pm)
**Treatment break***				
**Final dose****	172-176	PGE1<br>GM-CSF<br>PGE1	40 µg<br>75 µg/m²<br>40 µg	2 hours (9 am - 11 am)<br>4 hours (11 am - 3 pm)<br>2 hours (3 pm - 5 pm)

Legend: Experimental treatment protocol ogl. PGE1: Prostaglandine E1, GM-CSF: Granulocyte-Macrophage Colony-Stimulating Factor. i.v.: intravenous.

* prolonged by one week due to minor surgery

** repeated in five-day cycles from d194-198, d210-214, d224-228, slight differences in the intervals due to patient's preference

Figure S1: Frequencies of blasts and DC subtypes in patients’ blood in different stages of the disease and under different treatments.

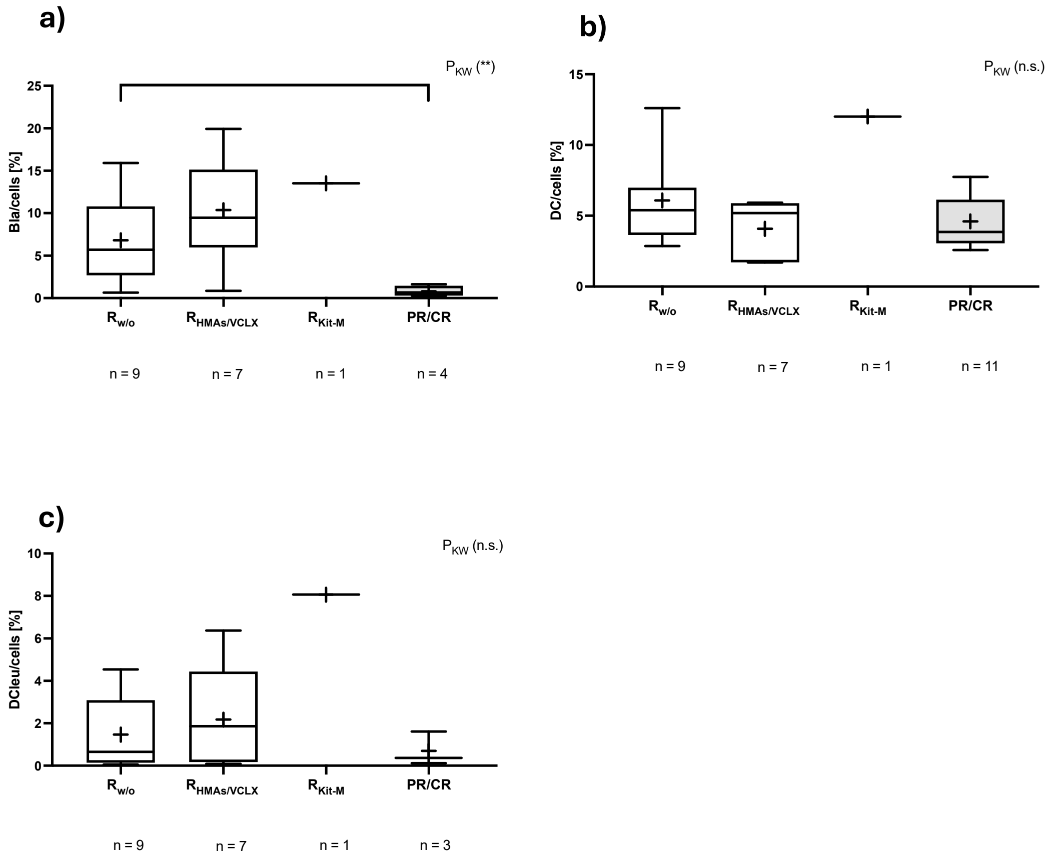

Legend to figure 1-7:
Given are median (—) and mean frequencies (+) ± standard deviation of cells and their subtypes in the groups ($R_{w/o}$, $R_{HMAs/VCLX}$, $R_{Kit\text{-}M}$, CR/PR). Explanations for treatment groups are given in table 4, abbreviations for cell subtypes are given in table 1c. Statistical analyses were conducted using the non-parametric Kruskal-Wallis (P_{KW}) test to compare all groups and the Mann-Whitney U-test to compare two groups. Differences were considered as highly significantly different with p-values ≤0.005 (***), as significantly different with p-values ≤0.05 (**) and as numerical trend with p-values ≤0.1 (*). Kit-M group (n=1) shown as individual, hypothesis-generating data, not as mean values

Figure S2: Frequencies of T cells and T cell subtypes in patients' blood in different stages of the disease and under different treatments.

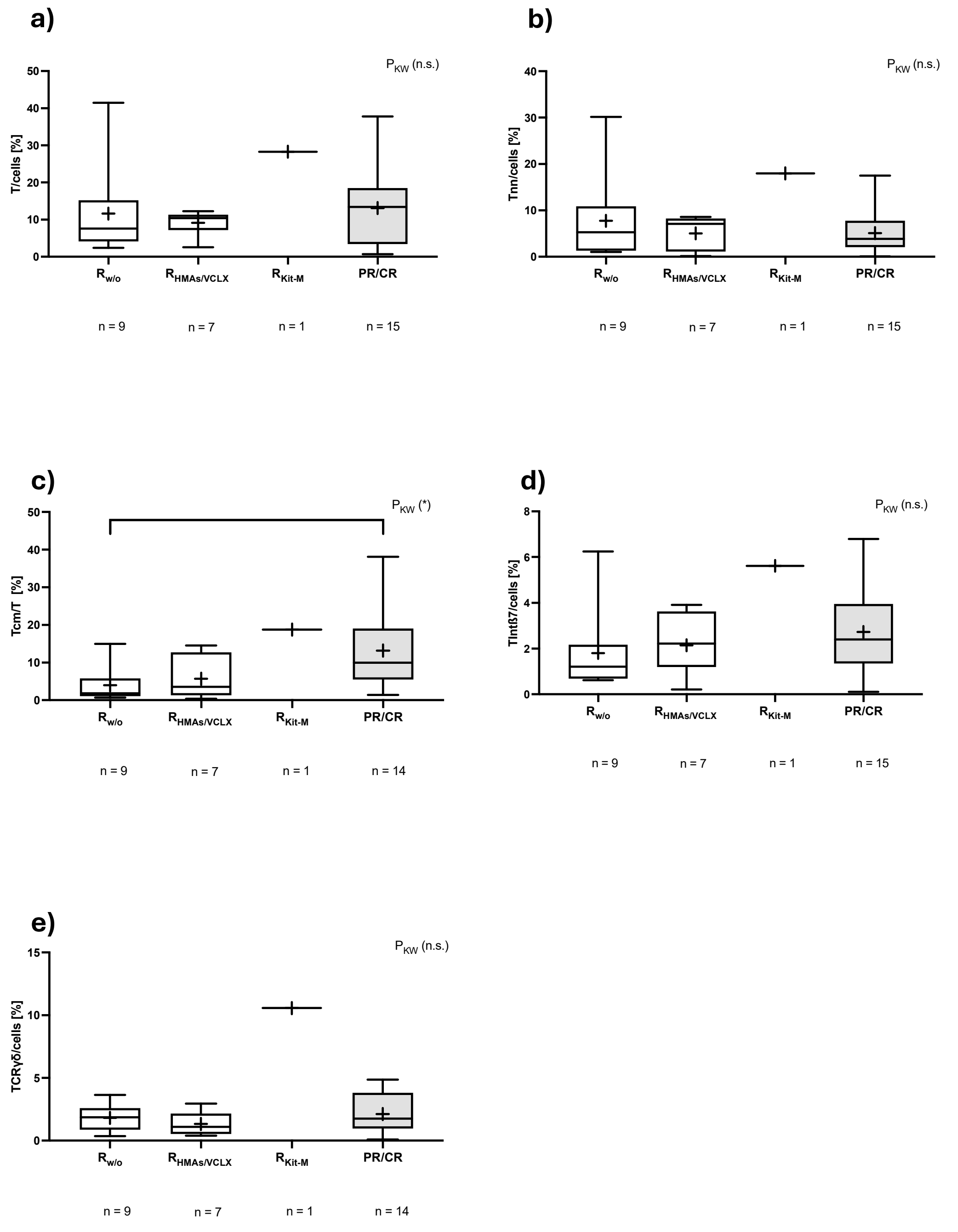

Figure S3: Frequencies of immune checkpoint expressing T cells in patients' blood in different stages of the disease and under different treatments.

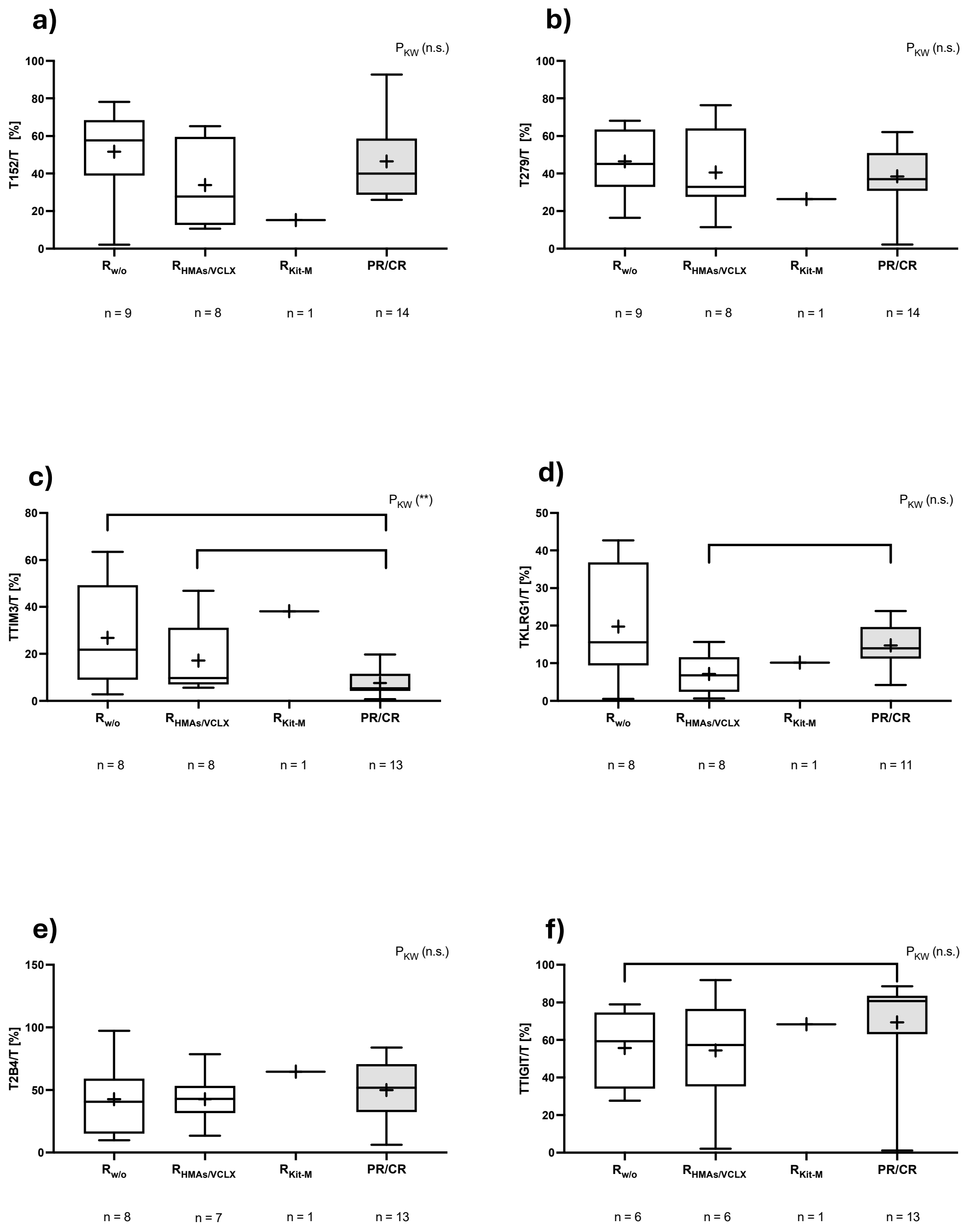

Figure S4: Frequencies of (potentially) leukemia specific T cells in patients' blood in different stages of the disease and under different treatments.

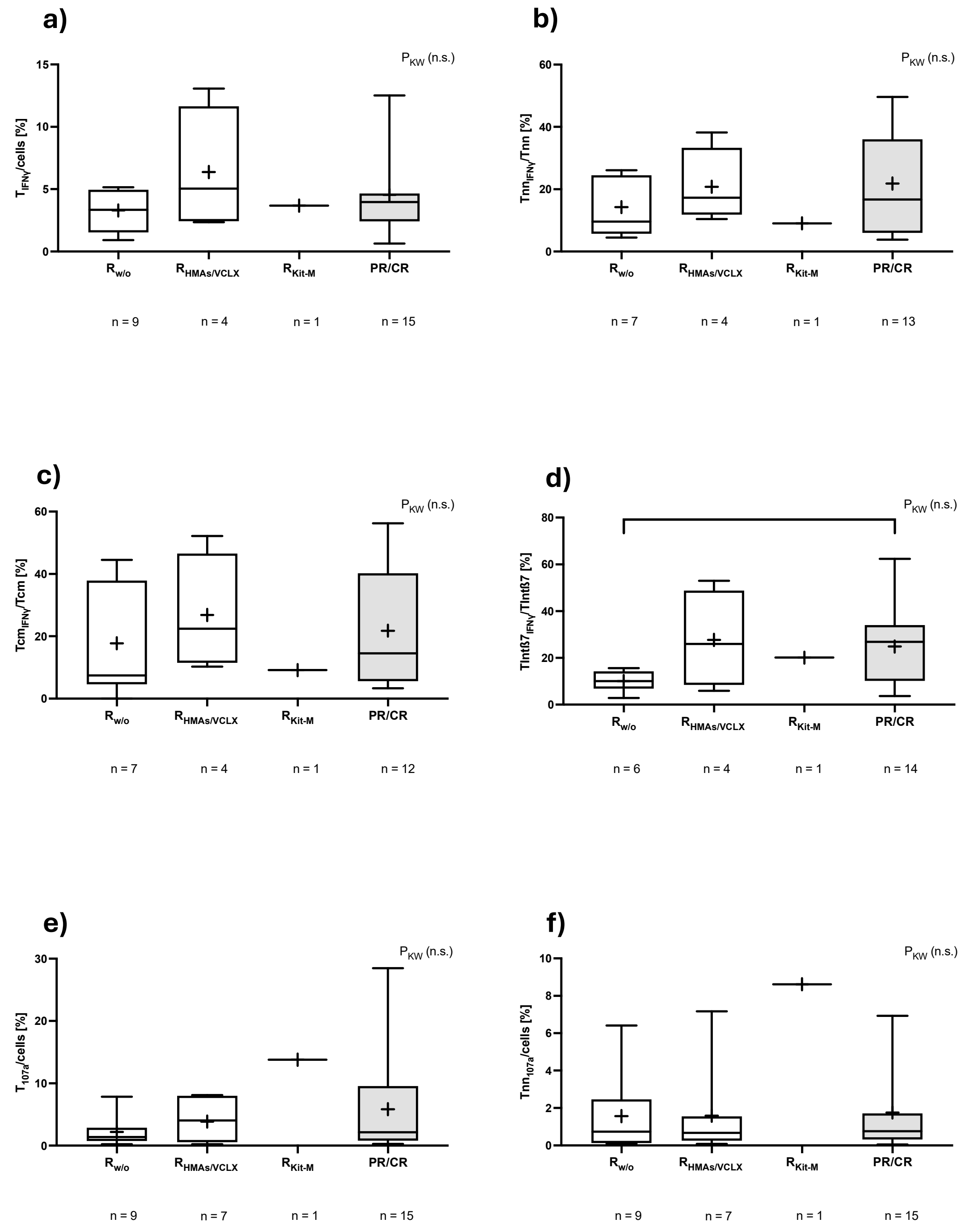

g)

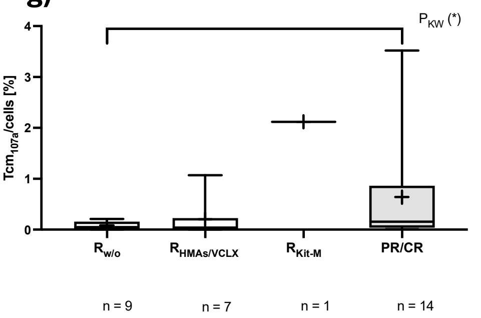

P KW (*)
Tcm 107a/cells [%]
0
1
2
3
4
R w/o
R HMAs/VCLX
R Kit-M
PR/CR
n = 9
n = 7
n = 1
n = 14

h)

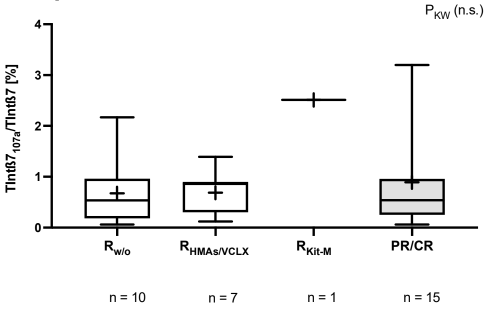

P KW (n.s.)
TIntß7 107a/TIntß7 [%]
0
1
2
3
4
R w/o
R HMAs/VCLX
R Kit-M
PR/CR
n = 10
n = 7
n = 1
n = 15

i)

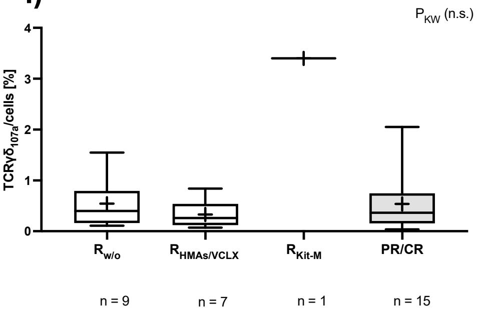

P KW (n.s.)
TCRγδ 107a/cells [%]
0
1
2
3
4
R w/o
R HMAs/VCLX
R Kit-M
PR/CR
n = 9
n = 7
n = 1
n = 15

Figure S5: Frequencies of innate immune cells in patients' blood in different stages of the disease and under different treatments.

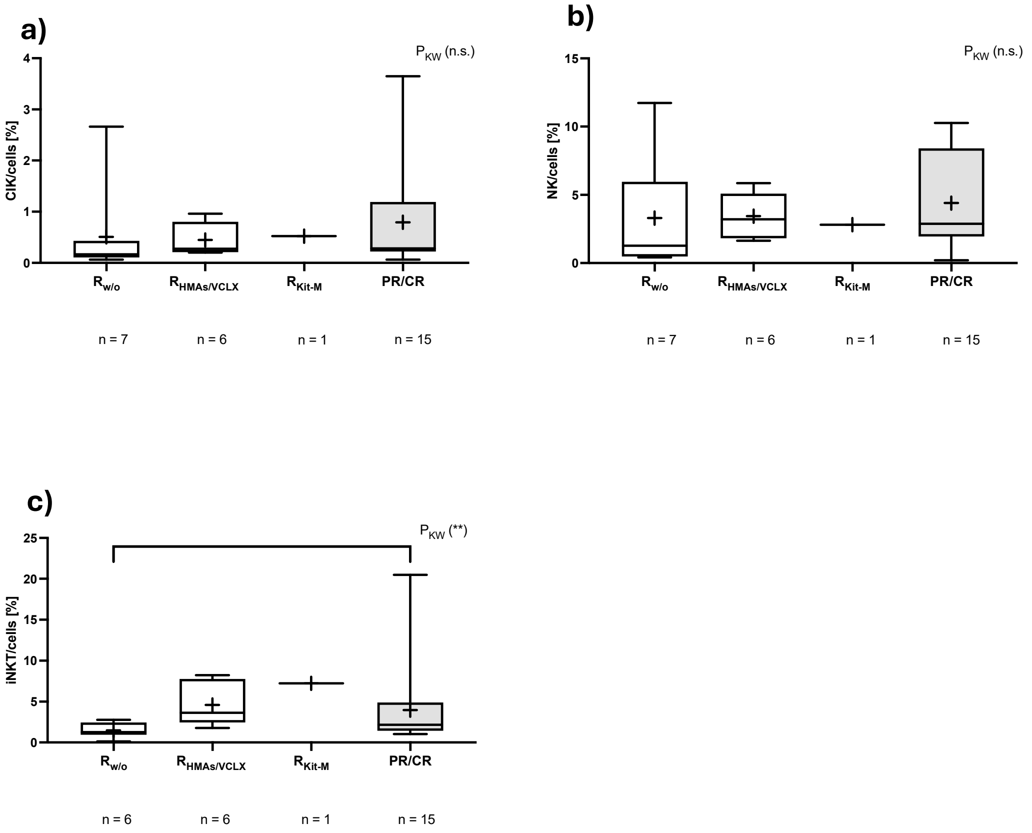

Figure S6: Frequencies of immune checkpointmarker expressing innate immune cells in patients' blood in different stages of the disease and under different treatments.

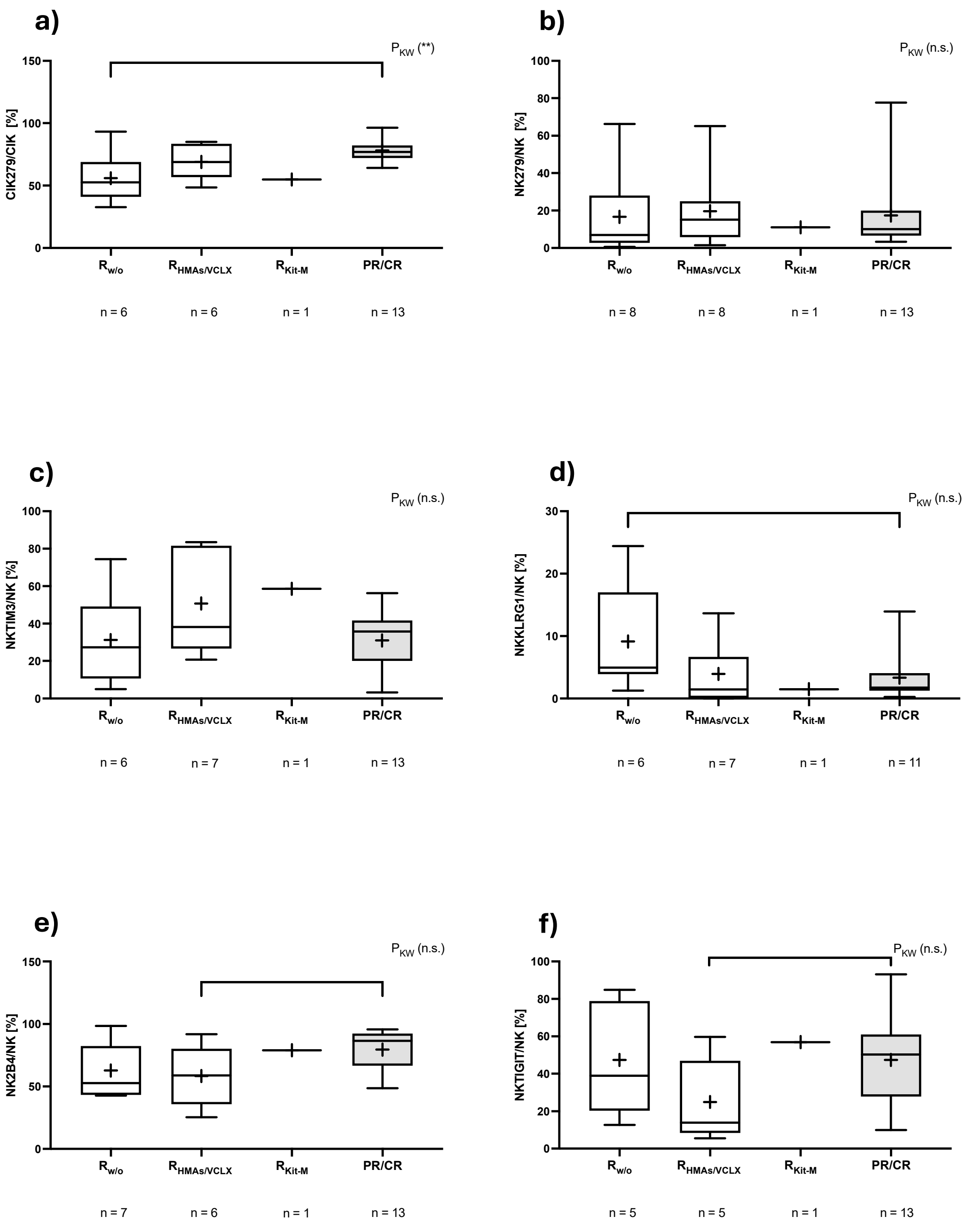

Figure S7: Frequencies of leukemia specific innate immune cells in patients' blood in different stages of the disease and under different treatments.

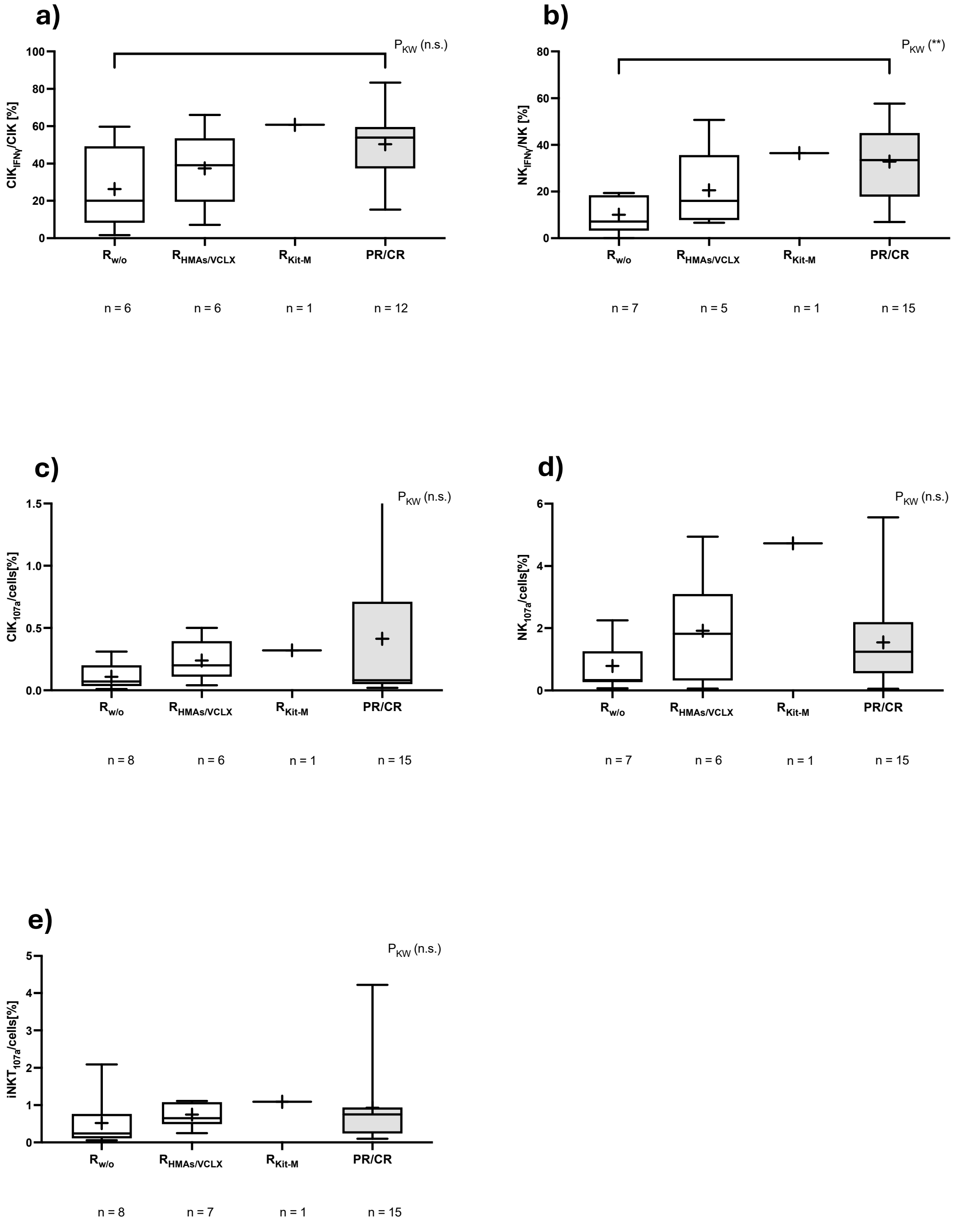

Legends are given below supp. figure 1

Figure S8: Gating strategy of (potentially leukemia specific) immune cells via Flowcytometry and analysis of immune-cell subsets

Immune cells were analysed using (isotype adapted) antibody panels (see below in supp table 1) designed for the quantification of immune cell subsets as given in table 1

Gating strategy for various immune subsets in a lymphocyte gate

Gating of lymphocytes in a SSC/FSC gate

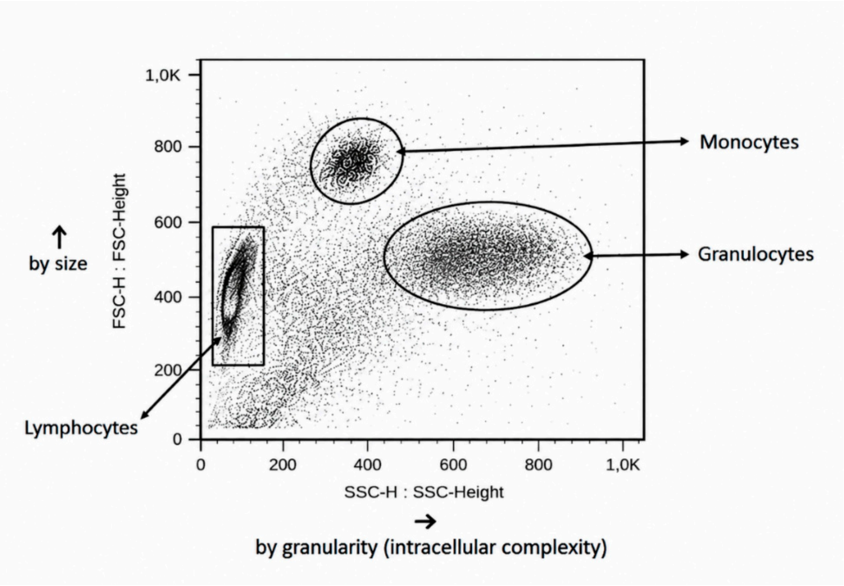

Gating strategy for T/NK and CIK cells

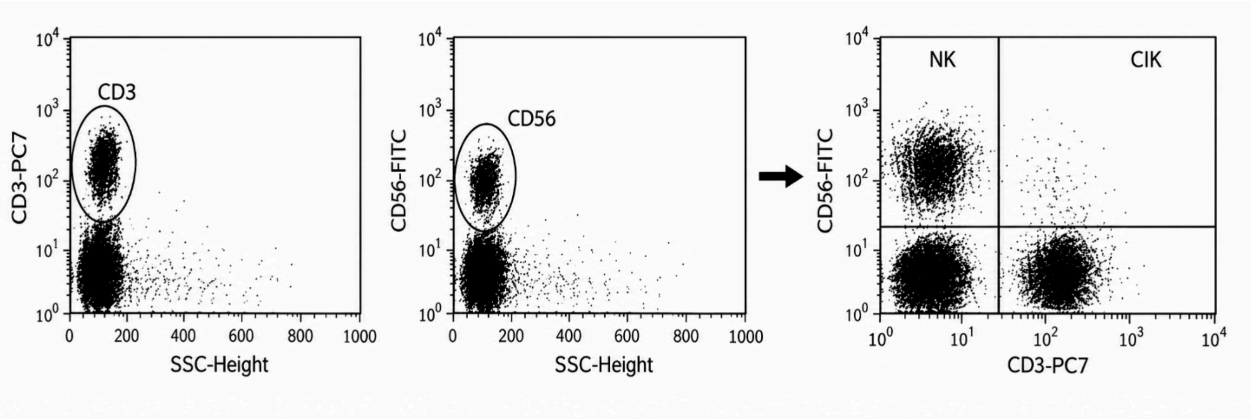

Gating strategy for Checkpoint marker (CD279, CD152) expressing T cells

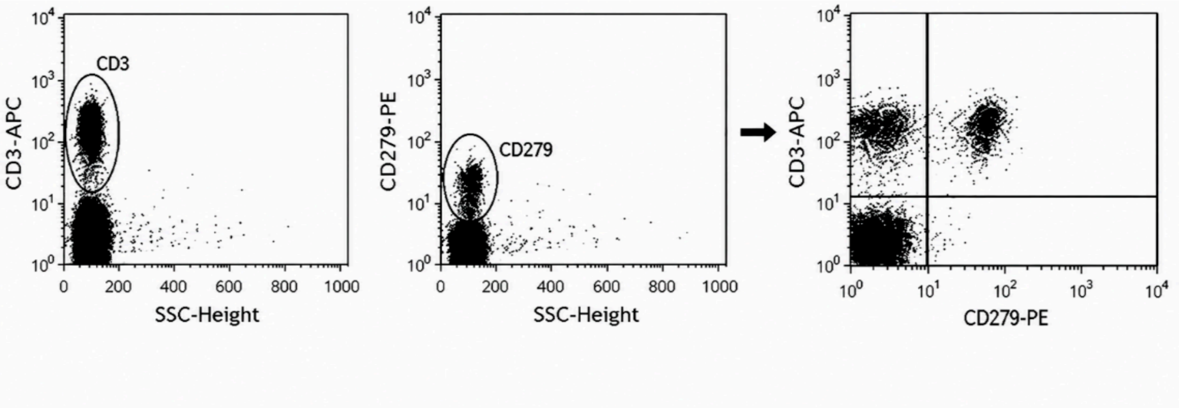

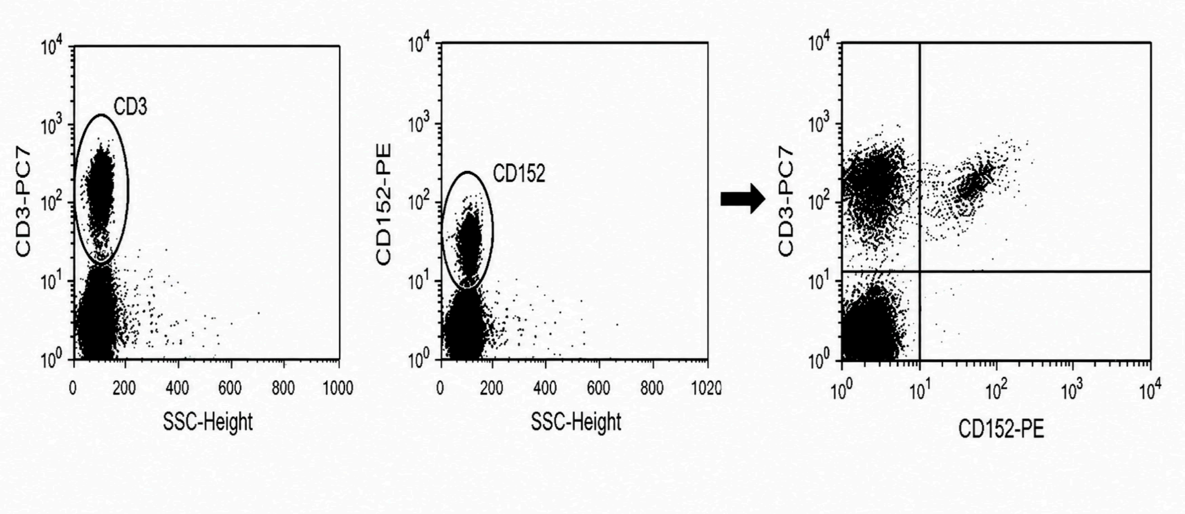

Gating strategy for degranulating (CD107a+) Tcells

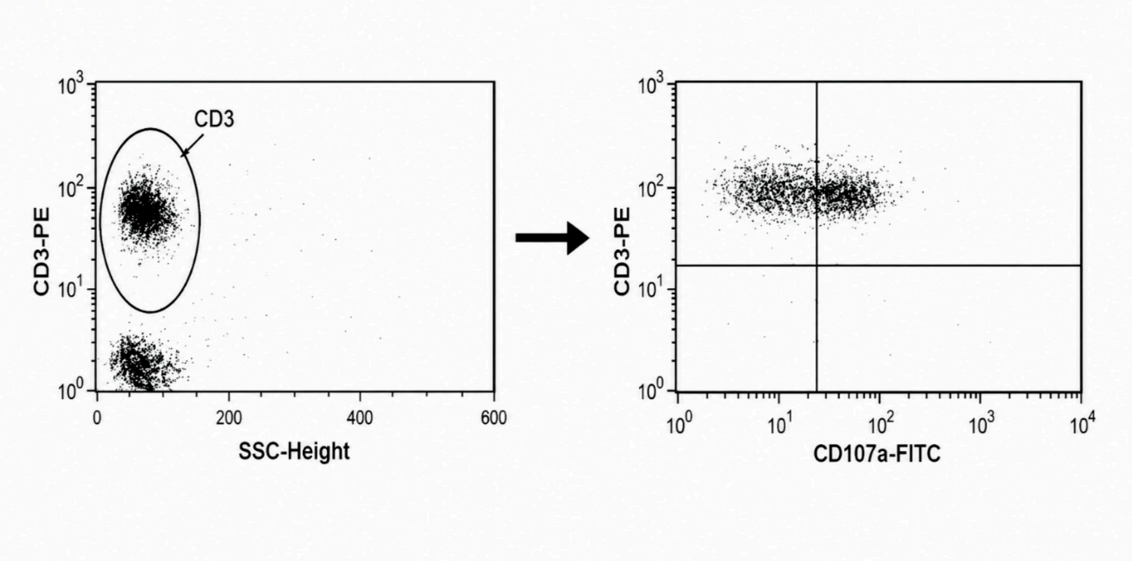

Gating strategy for intracellularly IFNγ producing T cells

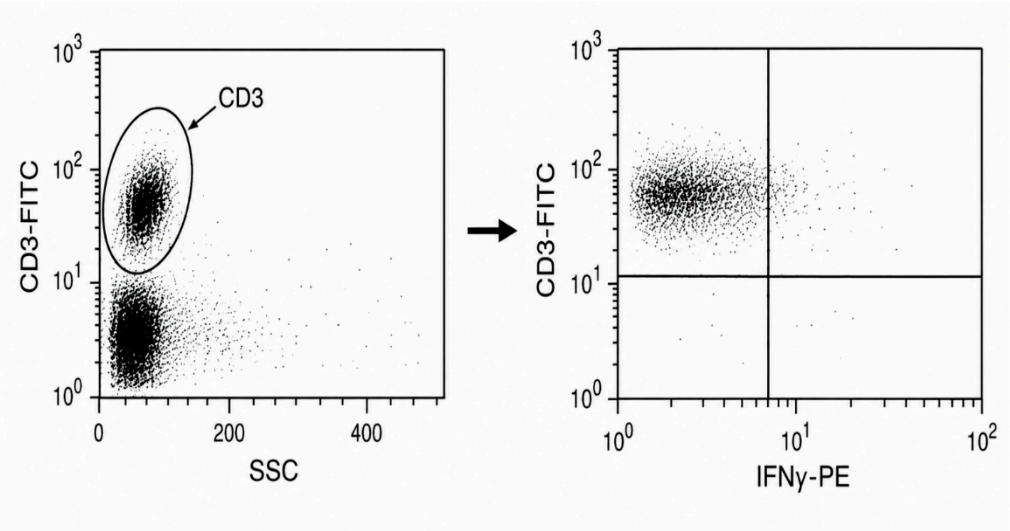

Table S2: PANELS USED FOR CELL SUBTYPE QUANTIFICATION

Panel 'Premeasurement': for a fast overall quantification of blasts, monocytes and lymphocytes: patient specific blast markers were included

Tube	FITC		PE		Pc7		APC	
1.	Isotyp	5,00 µl	Isotyp	5,00 µl	Isotyp	2,50 µl	Isotyp	2,50 µl
2.	-	5,00 µl	Bla	5,00 µl	Bla	2,50 µl	-	2,50 µl
3.	CD3	5,00 µl	CD56	5,00 µl	-	2,50 µl	-	2,50 µl

Panel ‚immune cell subsets

Tube	FITC		PE		PC7		APC	
1.	IgG1*	5,00 µl	IgG1*	5,00 µl	IgG1*	2,50 µl	IgG1*	2,50 µl
2.	-	5,00 µl	CD279	5,00 µl	Blast	2,50 µl	Blast	5,00 µl
3.	Blast	5,00 µl	CD152	5,00 µl	Blast	2,50 µl	-	5,00 µl
4.	Blast	5,00 µl	CD80	5,00 µl	-	5,00 µl	Blast	5,00 µl
5.	-	5,00 µl	CD279	5,00 µl	-	5,00 µl	CD3	2,50 µl
6.	-	5,00 µl	CD152	5,00 µl	CD3	5,00 µl	-	5,00 µl
8.	Blast	5,00 µl	TIM3	10,00 µl	CD56	5,00 µl	CD3	2,50 µl
9.	CD3	5,00 µl	-	5,00 µl	Blast	2,50 µl	TIM3	10,00 µl
10.	CD3	5,00 µl	-	5,00 µl	KLRG1	5,00 µl	CD56	2,50 µl
11.	CD56	5,00 µl	-	5,00 µl	CD3	5,00 µl	-	0,50 µl
12.	2B4	2,50 µl	-	5,00 µl	CD56	5,00 µl	CD3	2,50 µl
13.	CD56	5,00 µl	TIGIT	5,00 µl		5,00 µl	CD3	2,50 µl
14	CD56	5,00 µl	CD152	5,00 µl	CD3	2,50 µl	-	5,00 µl
15.	-	5,00 µl	CD279	5,00 µl	CD3	2,50 µl	CD56	2,50 µl
16.	Bla	5,00 µl	CD80	5,00 µl	Bla	2,50 µl	CD206	5,00 µl

Panel ‚intracellularly IFNg producing cells and degranulating (CD107a expressing) cells'

Tube	FITC		PE		PC7		APC	
1.	IgG_1	5,00µl	**IgG_1 IZ**	5,00µl	IgG_1	2,50µl	IgG_1	2,50µl
2.	-	5,00µl	**IFNγ**	2,50 µl	CD3	2,50µl	CD56	2,50µl
3.	CD3	5,00µl	**IFNγ**	2,50 µl	CD56	2,50µl	Int β7	2,50µl
4.	CD45Ro	5,00µl	**IFNγ**	2,50 µl	CD197	1,25µl	CD3	5,00µl

Tube	FITC		PE		PC7		APC	
1.	CD107a	1,25 µl	IgG_1	5,00 µl	IgG_1	2,50 µl	IgG_1	2,50 µl
2.	CD107a	1,25 µl	6b11	5,00 µl	CD3	2,50 µl	CD56	2,50 µl
3.	CD107a	1,25 µl	TCRγδ	2,50 µl	CD3	2,50 µl	-	2,50 µl
4.	CD107a	1,25 µl	CD127	5,00 µl	CD25	1,25 µl	CD4	2,50 µl
5.	CD107a	1,25 µl	CD3	5,00 µl	CD197	1,25 µl	CD45RO	2,50 µl
6.	CD107a	1,25 µl	CD56	5,00 µl	CD3	2,50 µl	Int. β7	2,50 µl
